# Systolic blood pressure as the mediator of the effect of early menarche on the risk of coronary artery disease: A Mendelian randomization study

**DOI:** 10.3389/fcvm.2022.1023355

**Published:** 2023-01-09

**Authors:** Hsien-Yu Fan, Yen-Tsung Huang, Yun-Yu Chen, Justin BoKai Hsu, Hung-Yuan Li, Ta-Chen Su, Hung-Ju Lin, Kuo-Liong Chien, Yang-Ching Chen

**Affiliations:** ^1^Institute of Epidemiology and Preventive Medicine, National Taiwan University, Taipei, Taiwan; ^2^Department of Family Medicine, Taipei Medical University Hospital, Taipei Medical University, Taipei, Taiwan; ^3^Institute of Statistical Science, Academia Sinica, Taipei, Taiwan; ^4^Department of Mathematics, National Taiwan University, Taipei, Taiwan; ^5^Department of Medical Research, Taichung Veterans General Hospital, Taichung, Taiwan; ^6^Cardiovascular Center, Taichung Veterans General Hospital, Taichung, Taiwan; ^7^Heart Rhythm Center, Division of Cardiology, Department of Medicine, Taipei Veterans General Hospital, Taipei, Taiwan; ^8^Cardiovascular Research Center, School of Medicine, National Yang Ming Chiao Tung University, Taipei, Taiwan; ^9^Department of Computer Science and Engineering, Yuan Ze University, Taoyuan, Taiwan; ^10^Department of Internal Medicine, National Taiwan University Hospital, Taipei, Taiwan; ^11^Department of Family Medicine, School of Medicine, College of Medicine, Taipei Medical University, Taipei, Taiwan; ^12^School of Nutrition and Health Sciences, College of Nutrition, Taipei Medical University, Taipei, Taiwan; ^13^Graduate Institute of Metabolism and Obesity Sciences, Taipei Medical University, Taipei, Taiwan

**Keywords:** menarche age, cardiovascular disease, metabolic factor, mediator, Mendelian randomization

## Abstract

**Background:**

Menarche timing may not be directly associated with the risk of coronary artery disease (CAD). Therefore, we investigated the roles of metabolic factors in explaining the effect of age at menarche on CAD risk.

**Methods:**

We identified women with age at menarche and CAD by using three analytical methods: Mendelian randomization (MR), logistic regression analysis, and Cox proportional hazard regression. The first two analyses were performed in the Taiwan Biobank (*N* = 71,923) study, and the last analysis was performed in the Chin-Shan Community Cardiovascular Cohort study (*N* = 1,598). We further investigated the role of metabolic factors in mediating the effect of age at menarche on CAD risk by using three complementary methods with mediation analyses.

**Results:**

One standard deviation of earlier age at menarche was associated with a 2% higher CAD risk [odds ratio = 1.02, 95% confidence interval (CI) = 1.001–1.03] in the MR analysis, an 11% higher risk (odds ratio = 1.11, 95% CI = 1.02–1.21) in the logistic regression analysis, and a 57% higher risk (hazard ratio = 1.57, 95% CI = 1.12–2.19) in the Cox proportional hazard regression. All the analyses consistently supported the role of systolic blood pressure in mediating this effect. The MR results indicated that 29% (95% CI = 26%–32%) of the effect of genetically predicted earlier age at menarche on CAD risk was mediated by genetically predicted systolic blood pressure.

**Conclusion:**

The results obtained using different analytical methods suggest that interventions aimed at lowering systolic blood pressure can reduce the cases of CAD attributable to earlier age at menarche.

## 1. Introduction

Cardiovascular diseases (CVDs), especially cerebrovascular accidents (CVAs) and coronary artery disease (CAD), are the leading causes of global death, estimating for more than 17 million deaths annually (1). The timing of menarche may play a causal role in the etiology of CVD (2, 3). Mishra et al. reported that each 1-year earlier age at menarche increased CAD risk by approximately 8% (2). However, the timing of menarche cannot be easily changed through any intervention. To determine the extent to which the adverse effects of earlier age at menarche can be mitigated by targeting its metabolic mediators, the extent of its effect on the risk of CVD mediated by metabolic factors should be examined. Some studies have investigated the body mass index (BMI)–mediated effects of age at menarche on the risks of hypertension and diabetes (4–7). However, no study has examined this effect on the risk of CVD. Moreover, the aforementioned studies have not quantified the role of other metabolic mediators (e.g., blood pressure, blood lipid, and glucose levels); this information is required to determine the appropriate clinical and public health interventions.

Observational studies focusing on a single mediator cannot comprehensively evaluate an individual’s lifetime exposure (8). For example, body measures performed at a single time point not only do not reflect changes across the life course but are also associated with measurement error bias due to diurnal variations, thus underestimating the mediation effect (8). Moreover, other biases (e.g., unmeasured confounders) may affect the results of observational studies (9). The Mendelian randomization (MR) approach uses genetic variants to investigate a causal relationship and has been applied to mediation analyses (10–14). The MR approach involves the random allocation of genetic variants at meiosis to infer a robust causal relationship against non-differential measurement error and confounding (10). Thus, mediation analysis through MR is less prone to bias due to measurement errors than conventional mediation analyses (13). A well-conducted MR for mediation analysis is valuable because it uses existing genetic data to improve causal inferences. Recent meta-analyses of genome-wide association studies have identified several menarche- and mediator-related genetic variants that can be used (15–19).

**FIGURE 1:**
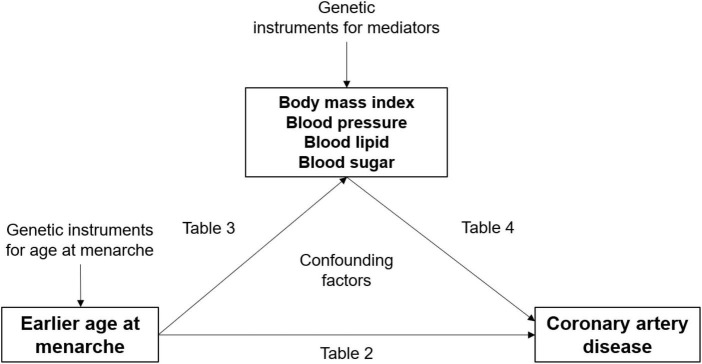
Observational and genetic associations/mediations investigated in the present study.

The MR approach has been adopted to investigate the causal effects of earlier age at menarche on metabolic factors and the causal effects of these metabolic factors on CAD risk (20–27). Although the results suggest that BMI, blood pressure, and blood glucose levels explain some of the causal effects of earlier age at menarche on CAD risk, they alone do not quantify the mediation effect. In the present study, we examined metabolic mediators in the effect of age at menarche on CAD risk by using three complementary methods: one-sample MR in the Taiwan Biobank (TWB) study, multivariable logistic regression in the TWB study, and multivariable Cox proportional hazard regression in the Chin-Shan Community Cardiovascular Cohort (CCCC) study. Studies (28, 29) have indicated that BMI, blood pressure, blood lipid levels, and blood glucose levels are affected by menarche timing and are risk factors for CAD. We hypothesized that metabolic factors mediate the effect of age at menarche on CAD risk (Figure 1). Elucidating the mechanisms between menarche timing and CAD can have powerful implications for CAD prevention and health promotion. It is therefore important to understand the clinical implications of changes to metabolic levels on inequalities in CAD risk.

## 2. Materials and methods

### 2.1. Population and study design

Observational data from the TWB study were used in MR and multivariable logistic regression analyses. The TWB study recruited 122,071 adults between 2008 and 2020 (30). The participants were administered questionnaires, and they underwent interviews, anthropometric and physical examinations, and blood sampling for biochemical analyses at the assessment centers. In the TWB study, we included 71,923 women with complete data on age at menarche, BMI, blood pressure, blood lipid and blood glucose levels, and CVD outcomes (Supplementary Figure S1-1). In the MR model, we included 64,734 participants with complete genotype data.

We also used data from the CCCC study in the multivariable Cox proportional hazard regression model. The CCCC study recruited the general population of the Chin-Shan Community in Taiwan aged ≥ 35 years between 1990 and 1991. All patients were followed up biennially between 1992 and 1995, between 1995 and 2000, and between 2000 and 2008 (31). Data collection was conducted through a questionnaire, physical examination, and blood biochemical analysis. In the CCCC study, the data of 1589 women without CAD outcomes at baseline (Supplementary Figure S1-1) and with complete information on age at menarche, BMI, blood pressure, blood lipid levels, blood glucose levels, and CAD outcomes were analyzed.

### 2.2. Outcomes

In the TWB study, CVD outcome data were collected through a questionnaire on family history, health status, medical diagnoses, and treatments. The participants were also asked whether a doctor had ever told them they had a certain CVD and its subtypes (e.g., CAD and CVA).

The endpoints in the CCCC study were incident CVD events during the follow-up period from 1990 to 2005. The CVD events considered in this study were CAD [defined as fatal CAD, non-fatal myocardial infarction, and coronary revascularization (32)] and CVA [defined as a sudden neurological deficit of vascular origin that lasted for more than 24 h (32, 33)].

### 2.3. Continuous exposure with continuous mediator variables

In the main analysis, we included continuous exposure with continuous mediator variables. Earlier age at menarche was included as the continuous exposure variable (Figure 1).

On the basis of the findings of previous studies (28, 29), we selected BMI, systolic blood pressure (SBP), diastolic blood pressure (DBP), total cholesterol (TC), high-density lipoprotein (HDL) cholesterol, low-density lipoprotein (LDL) cholesterol, and fasting blood glucose (FBG) levels as continuous mediator variables.

### 2.4. Genetic instruments

The MR approach relies on genetic variants satisfying three core assumptions of an instrumental variable. The underlying assumptions of MR are described in Supplementary material. On the basis of the findings of genome-wide association studies, we selected well-known single-nucleotide polymorphisms (SNPs) for each polygenetic score. Thus, the polygenetic score was associated only with age at menarche or the particular mediator of interest (Supplementary Table S1). After excluding pleiotropic SNPs (α = 0.05 divided by the number of SNPs), we selected 25 SNPs for earlier age at menarche and early menarche (Supplementary Tables S4, S5) (15); 18 SNPs for BMI and obesity (16), 8 SNPs for blood pressure and hypertension (17), 4 SNPs for blood lipid levels and dyslipidemia (18), and 10 SNPs for blood glucose levels and diabetes (19). The *F*-statistics for age at menarche and mediators were > 10 (Supplementary Table S1), indicating the presence of strong instrumental variables for each of our exposure of interest.

### 2.5. Statistical analyses

In mediation analysis, two models of relationships between variables are depicted: the total-effect model and the indirect-effect model (Supplementary Figure S1-2). The total effect is the effect of menarche timing on CAD risk, whereas a metabolic mediator is a variable that accounts for this effect. Furthermore, for mediation to occur, the indirect effect can be estimated. The proportion of the mediated effect was calculated by dividing the indirect effect by the total effect (11, 34). We used bootstrapping methods for significance testing in the mediation analysis (34). A more detailed description of the statistical methods is presented in Supplementary material.

#### 2.5.1. Effects of earlier age at menarche on the risk of CVD (total effects)

In the MR analysis, the effects of earlier age at menarche on CVD outcomes were investigated using two regression stages. The first stage involved the regression of age at menarche on its genetic instruments. Next, the predicted values of age at menarche and residuals from the first stage were used in the second stage of regression between the predicted values of age at menarche and CVD outcomes.

In the TWB study, we also performed multivariable logistic regression to estimate the association between earlier age at menarche and CVD outcomes.

In the CCCC study, multivariable Cox proportional hazard regression was performed to confirm the effect of earlier age at menarche on the 15-year risk of CVD outcomes.

All analyses were adjusted for the following potential confounders: age, education, current smoking, menopause, and regular physical activity.

#### 2.5.2. Mediation by metabolic factors (indirect effects)

We identified metabolic factors that serve as mediators of the effect of age at menarche on CAD risk because our three complementary approaches consistently suggested the total effect of earlier age at menarche on CAD risk.

The MR approach was adopted to estimate the effect of earlier age at menarche on each mediator. We performed regression-based multivariable MR to estimate the effect of each mediator on CAD risk after adjustment for the effect of the genetic instruments on age at menarche. The results of the two MR analyses were multiplied to estimate the indirect effect of age at menarche on CAD risk, which was mediated by the mediator of interest (11, 34).

In the TWB study, we also performed multivariable linear regression to estimate the effect of age at menarche on each mediator, with adjustments for confounders. Subsequently, we estimated the effect of each mediator on CAD risk, with additional adjustment for age at menarche. The two estimates were multiplied to estimate the indirect effect of earlier age at menarche through the mediator.

In the CCCC study, multivariable linear regression was used to estimate the effect of age at menarche on each mediator, with adjustments for confounders. We then performed multivariable Cox proportional hazard regression to confirm the effect of each mediator on the 15-year risk of CVD outcomes. The two estimates were multiplied to estimate the indirect effect of earlier age at menarche through the mediator.

#### 2.5.3. Mediation proportion

The mediation proportion was defined as the indirect effect divided by the total effect and considered “not causal” if the total and indirect effects were in opposite directions (35). Standard errors were derived through bootstrapping in the two cohort analyses.

### 2.6. Sensitivity analyses and external validation

One-sample MR analysis was performed using data from the TWB study. Complete details of the genetic instruments used are provided in Supplementary Table S1. Subsequently, we performed sensitivity analyses by using inverse-variance weighted, MR-Egger, median, and maximum likelihood methods (Supplementary Table S2). The sensitivity analyses were conducted by using the MR package implemented in R.

### 2.7. Statistical software and ethical approval

Statistical analyses were performed using R version 3.4.3 (The R Foundation for Statistical Computing). A detailed description of the statistical packages used is presented in Supplementary Table S2. The study protocol was approved by the Joint Institutional Review Board of Taipei Medical University (Number: N202107019) and the Research Ethics Committee of the National Taiwan University Hospital (Number: 202108099RINA).

## 3. Results

### 3.1. Demographic characteristics

Table 1 lists the characteristics of the participants in the TWB and CCCC studies. The number of participants varied among the different data sets according to the availability of exposures, outcomes, and covariates.

**TABLE 1:** General characteristics of participants in the Chin-Shan Community Cardiovascular Cohort and Taiwan Biobank.

Characteristic		Chin-Shan Community Cardiovascular Cohort study (*N* = 1,589)		Taiwan Biobank (*N* = 71,923)	
		**Mean or *N***	**SD or %**	**Mean or *N***	**SD or %**
Birth year	Years	1936	12.3	1965	11.0
Age at menarche	Years	15.8	1.8	13.2	1.4
	Earlier	392	24.7	20,338	28.3
	Later	1,197	75.3	51,585	71.7
Age	Years	53.3	12.0	50.2	10.7
	<65 years	1,279	80.5	66,015	91.8
	≥65 years	310	19.5	5,908	8.2
Menopause	Yes	642	42.3	34,675	48.2
Educational level	Low	994	62.6	4,521	6.3
	Medium	453	28.5	28,975	40.3
	High	142	8.9	38,407	53.4
Current smoker	Yes	82	5.2	7,435	10.3
Alcohol drinking	Yes	148	9.3	339	2.0
Regular physical activity	Yes	223	14.0	2,602	5.8
Sleep duration	Hours	6.9	1.4	6.7	1.1
Obesity	Yes	273	17.2	11,510	16.0
Dyslipidemia	Yes	112	7.0	4,629	7.1
Hypertension	Yes	233	14.7	7,353	19.3
Diabetes	Yes	188	11.8	3,199	6.7
Body mass index	kg⋅m^–2^	23.9	3.5	23.5	3.7
Systolic blood pressure	mmHg	125.8	21.2	116.0	17.8
Diastolic blood pressure	mmHg	77.0	11.1	70.6	10.3
Total cholesterol	mg⋅dL^–1^	202.1	44.8	197.5	36.0
Low-density lipoprotein cholesterol	mg⋅dL^–1^	141.6	43.8	120.3	31.8
High-density lipoprotein cholesterol	mg⋅dL^–1^	48.6	12.0	58.2	13.2
Fasting blood glucose	mg⋅dL^–1^	109.9	34.1	93.8	18.7

N, sample size; SD, standard deviation. The bold values represent the *p* value less than 0.05.

### 3.2. Effects of age at menarche on the risk of CVD (total effects)

MR analysis revealed the effect of earlier age at menarche on the risk of CVD (Table 2), with an odds ratio (OR) of 1.03 (95% CI = 1.01–1.05). In the logistic regression of the TWB study, one standard deviation of earlier age at menarche was associated with a higher risk of CVD [OR = 1.14; 95% confidence interval (CI) = 1.06–1.22].

**TABLE 2:** Effects of one standard deviation earlier age at menarche on the risk of cardiovascular disease.

Study	Outcome	Type of ratio	Estimation	95% CI	P
TWB-MR	CVD	Odds ratio	1.03	1.01–1.05	**0.02**
	CVA	Odds ratio	1.01	0.99–1.02	0.28
	CAD	Odds ratio	1.02	1.001–1.03	**0.04**
TWB-Observation	CVD	Odds ratio	1.14	1.06–1.22	**0.001**
	CVA	Odds ratio	1.18	1.05–1.33	**0.01**
	CAD	Odds ratio	1.11	1.02–1.21	**0.02**
CCCC	CVD	Hazard ratio	1.19	0.97–1.47	0.09
	CVA	Hazard ratio	1.03	0.79–1.33	0.84
	CAD	Hazard ratio	1.57	1.12–2.19	**0.01**

CAD, coronary artery disease; CCCC, the Chin-Shan Community Cardiovascular Cohort study; CI, confidence interval; CVA, cerebrovascular accident; CVD, cardiovascular disease; HR, hazard ratio; OR, odds ratio; TWB-MR, one-sample Mendelian randomization analysis in the Taiwan Biobank study; TWB-Observation, regression analysis in the Taiwan Biobank study; Standard deviation of age at menarche, 1.8 years in the prospective cohort study and 1.4 in the retrospective cohort and Mendelian randomization studies. The bold values represent the *p* value less than 0.05.

In the TWB study, the logistic regression indicated an association between earlier age at menarche and the risk of CVA (OR = 1.18; 95% CI = 1.05–1.32).

A robust association was noted between earlier age at menarche and CAD risk. In the MR study, the effect of earlier age at menarche was observed (OR = 1.02; 95% CI = 1.001–1.03). In the TWB study, the logistic regression indicated the effect of earlier age at menarche on CAD risk (OR = 1.11; 95% CI = 1.02–1.21). In the CCCC study, the Cox hazard regression model indicated that one standard deviation of earlier age at menarche was associated with a 57% higher risk of CAD, with a hazard ratio of 1.57 (95% CI = 1.12–2.19).

### 3.3. Mediation by metabolic factors (indirect effects)

In the MR analyses, earlier age at menarche was associated with higher SBP and DBP (Table 3), but only SBP level was associated with CAD risk (Table 4). For example, one standard deviation of earlier age at menarche was associated with a 3.15 mmHg higher SBP (95% CI = 0.53–5.69). One standard deviation of higher SBP was associated with a 4% higher risk of CAD (OR = 1.04, 95% CI = 1.001–1.09).

**TABLE 3:** Effects of one standard deviation earlier age at menarche on metabolic mediator.

Study	Metabolic mediator	Regression coefficients	95% CI	*P*
TWB-MR	BMI (kg⋅m^–2^)	0.52	−0.08 to 1.09	0.09
	SBP (mmHg)	3.15	0.53 to 5.69	**0.02**
	DBP (mmHg)	2.13	0.48 to 3.72	**0.01**
	TC (mg⋅dL^–1^)	−1.94	−7.57 to 3.52	0.50
	LDL (mg⋅dL^–1^)	−2.81	−7.92 to 2.13	0.28
	HDL (mg⋅dL^–1^)	0.07	−2.08 to 2.16	0.95
	FBG (mg⋅dL^–1^)	2.12	−0.85 to 4.99	0.16
TWB-Observation	BMI (kg⋅m^–2^)	0.58	0.55 to 0.61	**0.2 × 10^–15^**
	SBP (mmHg)	1.02	0.89 to 1.15	**0.2 × 10^–15^**
	DBP (mmHg)	0.62	0.54 to 0.70	**0.2 × 10^–15^**
	TC (mg⋅dL^–1^)	0.46	0.18 to 0.73	**0.001**
	LDL (mg⋅dL^–1^)	0.66	0.41 to 0.90	**0.3 × 10^–6^**
	HDL (mg⋅dL^–1^)	−0.60	−0.70 to−0.49	**0.2 × 10^–15^**
	FBG (mg⋅dL^–1^)	0.61	0.46 to 0.75	**0.2 × 10^–15^**
CCCC	BMI (kg⋅m^–2^)	0.48	0.30 to 0.65	**0.1 × 10^–6^**
	SBP (mmHg)	1.11	0.25 to 1.95	**0.01**
	DBP (mmHg)	0.63	0.13 to 1.11	**0.01**
	TC (mg⋅dL^–1^)	0.17	−1.74 to 2.02	0.86
	LDL (mg⋅dL^–1^)	0.45	−1.34 to 2.18	0.62
	HDL (mg⋅dL^–1^)	−0.78	−1.39 to−0.19	**0.01**
	FBG (mg⋅dL^–1^)	0.44	−1.13 to 1.96	0.58

BMI, body mass index; CCCC, the Chin-Shan Community Cardiovascular Cohort study; CI, confidence interval; DBP, diastolic blood pressure; FBG, fasting blood glucose; HDL, high-density lipoprotein cholesterol; HR, hazard ratio; LDL, low-density lipoprotein cholesterol; OR, odds ratio; SBP, systolic blood pressure; TC, total cholesterol; TWB-MR, one-sample Mendelian randomization analysis in the Taiwan Biobank study; TWB-Observation, regression analysis in the Taiwan Biobank study; Standard deviation of the exposure and mediators in the prospective cohort study, 1.8 years for age at menarche, 3.5 kg⋅m^–2^ for BMI, 21.2 mmHg for SBP, 11.1 mmHg for DBP, 44.8 mg⋅dL^–1^ for TC, 43.8 mg⋅dL^–1^ for LDL, 12 mg⋅dL^–1^ for HDL, and 34.1 mg⋅dL^–1^ for FBG; Standard deviation of the exposure and mediators in the retrospective cohort and Mendelian randomization studies, 1.4 years for age at menarche, 3.7 kg⋅m^–2^ for BMI, 17.8 mmHg for SBP, 10.3 mmHg for DBP, 36 mg⋅dL^–1^ for TC, 31.8 mg⋅dL^–1^ for LDL, 13.2 mg⋅dL^–1^ for HDL, and 18.7 mg⋅dL^–1^ for FBG. The bold values represent the *p* value less than 0.05.

**TABLE 4:** Effects of one standard deviation mediator on the risk of coronary artery disease.

Study	Metabolic mediator	Type of ratio	Estimation	95% CI	*P*
TWB-MR	BMI (kg⋅m^–2^)	Odds ratio	1.03	1.003–1.06	**0.03**
	SBP (mmHg)	Odds ratio	1.04	1.001–1.09	**0.04**
	DBP (mmHg)	Odds ratio	1.06	0.99–1.14	0.07
	TC (mg⋅dL^–1^)	Odds ratio	1.01	0.99–1.02	0.05
	LDL (mg⋅dL^–1^)	Odds ratio	1.01	1.005–1.02	**0.005**
	HDL (mg⋅dL^–1^)	Odds ratio	1.002	0.991–1.01	0.73
	FBG (mg⋅dL^–1^)	Odds ratio	1.03	0.998–1.07	0.07
TWB-Observation	BMI (kg⋅m^–2^)	Odds ratio	1.003	1.003–1.004	**0.2 × 10^–15^**
	SBP (mmHg)	Odds ratio	1.002	1.001–1.002	7.1 **× 10^–5^**
	DBP (mmHg)	Odds ratio	1.0001	0.99–1.001	0.77
	TC (mg⋅dL^–1^)	Odds ratio	0.995	0.995–0.997	**0.2 × 10^–15^**
	LDL (mg⋅dL^–1^)	Odds ratio	0.995	0.995–0.997	**0.2 × 10^–15^**
	HDL (mg⋅dL^–1^)	Odds ratio	0.997	0.997–0.998	**8.4 × 10^–14^**
	FBG (mg⋅dL^–1^)	Odds ratio	1.004	1.003–1.004	**0.2 × 10^–15^**
CCCC	BMI (kg⋅m^–2^)	Hazard ratio	1.69	1.23–2.33	**0.001**
	SBP (mmHg)	Hazard ratio	2.69	1.84–3.95	**3.4 × 10^–7^**
	DBP (mmHg)	Hazard ratio	2.52	1.81–3.52	**4.7 × 10^–8^**
	TC (mg⋅dL^–1^)	Hazard ratio	1.49	0.99–2.25	0.05
	LDL (mg⋅dL^–1^)	Hazard ratio	1.64	1.08–2.50	**0.02**
	HDL (mg⋅dL^–1^)	Hazard ratio	0.65	0.42–1.02	0.06
	FBG (mg⋅dL^–1^)	Hazard ratio	1.37	1.08–1.73	**0.009**

BMI, body mass index; CCCC, the Chin-Shan Community Cardiovascular Cohort study; CI, confidence interval; DBP, diastolic blood pressure; FBG, fasting blood glucose; HDL, high-density lipoprotein cholesterol; HR, hazard ratio; LDL, low-density lipoprotein cholesterol; OR, odds ratio; SBP, systolic blood pressure; TC, total cholesterol; TWB-MR, one-sample Mendelian randomization analysis in the Taiwan Biobank study; TWB-Observation, regression analysis in the Taiwan Biobank study; Standard deviation of the exposure and mediators in the prospective cohort study, 1.8 years for age at menarche, 3.5 kg⋅m^–2^ for BMI, 21.2 mmHg for SBP, 11.1 mmHg for DBP, 44.8 mg⋅dL^–1^ for TC, 43.8 mg⋅dL^–1^ for LDL, 12 mg⋅dL^–1^ for HDL, and 34.1 mg⋅dL^–1^ for FBG; Standard deviation of the exposure and mediators in the retrospective cohort and Mendelian randomization studies, 1.4 years for age at menarche, 3.7 kg⋅m^–2^ for BMI, 17.8 mmHg for SBP, 10.3 mmHg for DBP, 36 mg⋅dL^–1^ for TC, 31.8 mg⋅dL^–1^ for LDL, 13.2 mg⋅dL^–1^ for HDL, and 18.7 mg⋅dL^–1^ for FBG. The bold values represent the *p* value less than 0.05.

In the TWB study, the regression models supported SBP as the critical mediator in the relationship between age at menarche and CAD risk. One standard deviation of earlier age at menarche was associated with a 1.02 mmHg higher SBP (95% CI = 0.89–1.15). One standard deviation of higher SBP was associated with a 0.2% higher risk of CAD (OR = 1.002, 95% CI = 1.001–1.002).

Similar to the above findings, the Cox hazard regression model also supported SBP as a critical mediator in the relationship between age at menarche and CAD risk. One standard deviation of earlier age at menarche was associated with a 1.11 mmHg higher SBP (95% CI = 0.25–1.95). One standard deviation of higher SBP was associated with a higher risk of CAD, with a hazard ratio of 2.69 (95% CI = 1.84–3.95).

### 3.4. Proportion mediated

**FIGURE 2:**
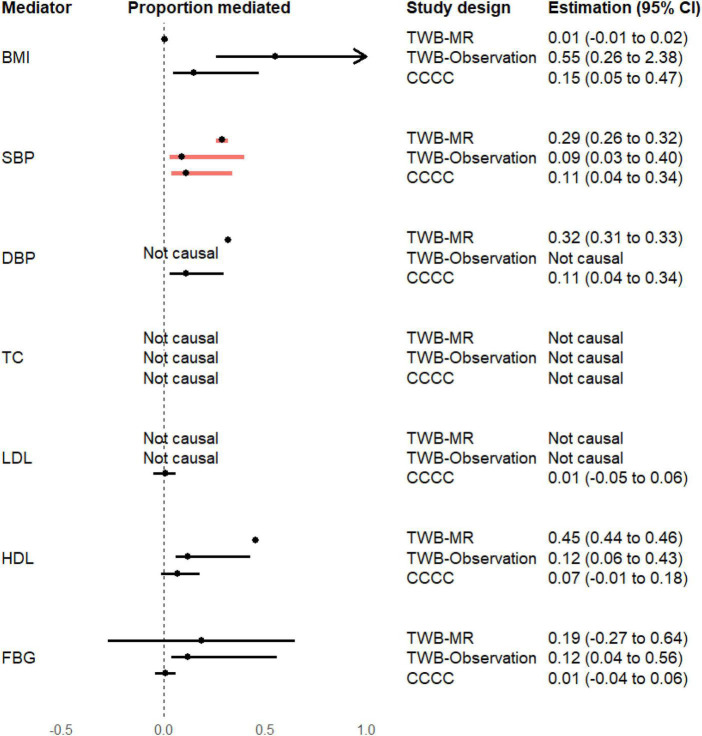
Proportion of the mediation effects of earlier age at menarche on the risk of coronary artery disease. BMI, body mass index; CCCC, the Chin-Shan Community Cardiovascular Cohort study; CI, confidence interval; DBP, diastolic blood pressure; FBG, fasting blood glucose; HDL, high-density lipoprotein cholesterol; LDL, low-density lipoprotein cholesterol; SBP, systolic blood pressure; TC, total cholesterol; TWB-MR, one-sample Mendelian randomization analysis in the Taiwan Biobank study; TWB-Observation, regression analysis in the Taiwan Biobank study. The proportion of mediation effect is defined as “not causal” if the total and indirect effects are in opposite directions.

As presented in Figure 2, in the MR analysis, the proportion of the effect of earlier age at menarche on CAD risk mediated by SBP, DBP, and HDL cholesterol was 0.29 (95% CI = 0.26–0.32), 0.32 (95% CI = 0.31–0.33), and 0.45 (95% CI = 0.44–0.46), respectively.

In the TWB observational analysis, the proportion of the effect of earlier age at menarche on CAD risk mediated by BMI, SBP, and DBP was 0.15 (95% CI = 0.05–0.47), 0.11 (95% CI = 0.04–0.34), and 0.11 (95% CI = 0.03–0.30), respectively.

In the CCCC study, the proportion of the effect of earlier age at menarche on CAD risk mediated by BMI, SBP, HDL cholesterol, and FBG was 0.55 (95% CI = 0.26–2.38), 0.09 (95% CI = 0.03–0.40), 0.12 (95% CI = 0.06–0.43), and 0.12 (95% CI = 0.04–0.56), respectively.

All analysis methods consistently supported the role of SBP in mediating this effect. The MR results indicated that the high proportion (29%; 95% CI = 26–32%) of the effect of genetically predicted earlier age at menarche on CAD risk was mediated by genetically predicted SBP.

### 3.5. Sensitivity analyses and external validation

As shown in Supplementary Table S3, the sensitivity analysis yielded comparable results. This additional analysis produced less precise estimates and greater confidence intervals. The inaccuracy in the one-sample MR approach indicated the need for considerably large sample sizes to realize adequate power when estimating mediation in the MR approach.

## 4. Discussion

### 4.1. Main findings

To the best of our knowledge, this is the first study to consider metabolic factors as mediators of the effect of earlier age at menarche on CAD risk. We used the MR approach to quantify the proportion of the effect of earlier age at menarche on CAD risk mediated by different metabolic mediators. We observed that the increased risk of CAD caused by earlier age at menarche was partly mediated by elevated SBP. However, BMI, DBP, and blood lipid and FBG levels did not appear to be causal mediators. Our data provide crucial information because CAD is a growing health problem worldwide, partly because of the decline in age at menarche. Although menarche timing is an early-life factor, it is not easily modifiable. Thus, modifying metabolic mediators can be an alternative approach to reducing CAD risk. Our results indicated that the majority of the effect of earlier age at menarche on CAD risk was mediated by hypertension-related traits. In our study, one-fourth of the women with earlier menarche met the criteria for hypertension diagnosis. SBP was the most notable mediator and accounted for a quarter of the effect. Our finding supports early identification of hypertension and the well-controlled blood pressure in adolescent women with early menarche can reduce the further risk of CAD in later life.

### 4.2. Findings in context

Several studies have used observational multivariable regression methods to determine the mediating role of body composition–related factors in the effect of earlier age at menarche on metabolic cardiovascular risk factors (4, 6, 29). A Brazilian study by Bubach and colleagues reported that BMI explained 47% of the effect of earlier age at menarche on DBP (29). Similarly, Werneck and colleagues indicated that BMI mediated the association between age at menarche and blood pressure (4). A Chinese study by Zhang et al. reported that BMI mediated the association of age at menarche not only with hypertension (6) but also with diabetes (5). However, they did not examine the mediating role of these metabolic factors in the effect of age at menarche on CVD outcomes, particularly CAD. The current study provides crucial research advances. Our findings revealed that SBP mediated the association of age at menarche with CAD. Earlier age at menarche is a typical feature of early vascular aging observed in adults with primary hypertension (36, 37), which is associated with accelerated biological development and neuroimmunometabolic abnormalities (37). Primary hypertension in childhood is not only an early vascular aging event but also a risk factor for CAD.

Several biological mechanisms have been proposed to explain the effect of earlier age at menarche on the risk of CVD. Menarche timing reflects an overlap of SNPs with obesity-related traits, height, diabetes, and pubertal anthropometrics. Luijken and colleagues suggested that the association of earlier age at menarche with CVD risk can be explained by two mechanisms (28). First, earlier age at menarche can lead to higher BMI, which is frequently described as a risk factor for CVD. Second, earlier age at menarche can lead to shorter height, which has been reported to be a risk factor for CAD, similar to SBP and BMI. Therefore, not only body height but also SBP and BMI may be involved in the effect of earlier age at menarche on CAD risk. Our three approaches indicated that SBP mediated the effect of earlier age at menarche on CAD risk. The difference in the diameters of vessels and coronary arteries is another mechanism that can explain the effect of earlier age at menarche on CAD risk. While Paajanen and colleagues suggested that short stature is a risk factor for CAD through the effect of small coronary arteries or blood vessels, our findings suggest smaller coronary arteries may be occluded in adolescent women under similar mechanisms (38). Future studies should investigate whether the height in adults and the coronary artery diameter mediate the effect of earlier age at menarche on CAD risk.

### 4.3. Strengths and limitations

The major strength of our study is that the use of multiple data sources and multiple complementary methods, each with different types of biases, improved the accuracy of our findings. Furthermore, we used the MR approach for the random allocation of genetic variants to improve causal inference. The potential limitation of our study is the imprecision in the one-sample MR analysis, which indicated the need for an ideal sample size to realize adequate power when estimating each effect in the causal inference.

The CCCC study is small and not representative of the general population. In addition, the results of the retrospective cohort study may be limited by reverse causation. For example, preexisting illness and weight gain may cause reverse causation bias. However, the use of genetic markers as instrumental variables can describe random fluctuations in the number of gene variants in this population, thereby mitigating the potential bias. The genetic markers temporally precede CVD outcomes and provided some immunity to reverse causation. Although MR approaches have been widely used to investigate the effect of obesity-related traits on cardiovascular outcomes (39), the relationship between obesity and cardiovascular outcomes may be bidirectional. In our analyses, in which we focused on the direction from metabolic traits to cardiovascular outcomes, the use of many robust measures for metabolic factors made it unlikely that the causal inferences derived from MR were limited due to reverse causation.

Another limitation is that MR estimates might be limited by pleiotropic effects, in which instruments exhibit effects on the outcome through pathways other than the exposure pathway. Given the potential for genetic pleiotropy, we also performed MR sensitivity analyses, which are more robust to such pleiotropy; the analyses generated results consistent with those of the one-sample MR analyses. If the use of genetic markers as instrumental variables had a monotonic effect on the menarche timing and mediators, our MR estimates reflected the average effect of menarche timing and mediators on CVD outcomes for all participants whose menarche timing or mediators were influenced by genetic markers. We obtained little evidence of the heterogeneity of the genetic effect on the menarche timing/metabolic mediators. The effects of genetic variants on menarche timing or mediators may be similar across the population; in this case, the MR inference may be an unbiased estimation of the average effect on this population. The use of binary exposures (including mediators) may underestimate indirect effects and an overestimation of direct effects. Finally, our study lacked the power to obtain precise MR estimates, including the sensitivity analyses. These MR estimates may not evidence an association for any of the studied mediators or the effects on the outcome studied. Therefore, we used the statements for the MR results because there is no complete power to infer causality.

The TWB included Asian participants, which might limit the generalizability of our results to other populations and ethnicities. The one-sample analysis was not representative of all populations and subject to healthy volunteer bias. For example, volunteers were less likely to be hypertensive and more likely to have a lower risk of CAD than their non-volunteer counterparts. Additional studies on this topic are warranted.

### 4.4. Clinical and public health implications

Our findings have clear applications and implications for practice. Given the effect of earlier age at menarche on CAD risk, early interventions that aimed at lowering blood pressure, even in early life, need to be promoted. In addition, considering that SBP mediates the association between age at menarche and CAD, interventions focusing on women with early hypertension need to be implemented. Furthermore, higher SBP was associated with higher CAD risk; thus, interventions aimed at preventing hypertension throughout the lifespan should be incorporated as part of strategies to reduce CAD risk in women.

## 5. Conclusion

Our findings, obtained using different analytical methods, including genetic markers that can draw a causal relationship, suggest that interventions aimed at lowering SBP can reduce CAD risk in women with earlier age at menarche. However, approximately three-fourth of the effect of earlier age at menarche on CAD risk was not mediated by SBP, necessitating additional studies to identify the other mediators. Future studies should also assess two or more sequential mediators in the relationship between age at menarche and CADs.

## Data availability statement

The raw data supporting the conclusions of this article will be made available by the authors, without undue reservation.

## Ethics statement

The studies involving human participants were reviewed and approved by the Joint Institutional Review Board of Taipei Medical University (Number: N202107019) and the Research Ethics Committee of the National Taiwan University Hospital (Number: 202108099RINA). The patients/participants provided their written informed consent to participate in this study.

## Author contributions

H-YF contributed to the data analyses, interpretation of data, and writing of the manuscript. Y-TH assisted in the statistical analysis and data interpretation and revised the manuscript critically for important intellectual content. Y-YC contributed to the analyses and interpretation of data from the CCCC study. JH assisted in the analyses and interpretation of genetic data. H-YL contributed to interpretation of data and critically revised the manuscript for intellectual content. T-CS, H-JL, and K-LC contributed to the CCCC data collection and interpretation. K-LC and Y-CC reviewed the study design, assisted in data acquisition and interpretation, supervised the study, and revised the manuscript critically for important intellectual content. All authors approved the final manuscript as submitted, published, and agreed to be accountable for all aspects of the work.
