# Corrigendum: Systolic blood pressure as the mediator of the effect of early menarche on the risk of coronary artery disease: A Mendelian randomization study

**DOI:** 10.3389/fcvm.2023.1165387

**Published:** 2023-03-01

**Authors:** Hsien Yu Fan, Yen-Tsung Huang, Yun-Yu Chen, Justin BOKAI HSU, Hung-Yuan Li, Ta-Chen Su, Hung-Ju Lin, Kuo-Liong Chien, Yang Ching Chen

**Affiliations:** ^1^ Institute of Epidemiology and Preventive Medicine, College of Public Health, National Taiwan University, Taipei, Taiwan; ^2^ Institute of Statistical Science, Academia Sinica, Taipei, Taiwan; ^3^ Department of Medical Research, Taichung Veterans General Hospital, Taichung, Taiwan; ^4^ Department of Computer Science and Engineering, Yuan Ze University, Taoyuan, Taiwan; ^5^ Department of Internal Medicine, National Taiwan University Hospital, Taipei, Taiwan; ^6^ Department of Family Medicine, School of Medicine, College of Medicine, Taipei Medical University, Taipei, Taiwan

**Keywords:** menarche age, cardiovascular disease, metabolic factor, mediator, Mendelian randomization

**A Corrigendum on**
Systolic blood pressure as the mediator of the effect of early menarche on the risk of coronary artery disease: A Mendelian randomization study By Fan H-Y, Huang Y-T, Chen Y-Y, Hsu JB, Li H-Y, Su T-C, Lin H-J, Chien K-L and Chen Y-C (2023) Front. Cardiovasc. Med. 9:1023355. doi: 10.3389/fcvm.2022.1023355

**Incorrect Funding**

In the published article, there was a mistake in the Funding statement. One grant funding source of this research was missed, which is “Taipei Medical University TMU108-AE1-B34”. The correct Funding statement appears below.

The authors apologize for this error and state that this does not change the scientific conclusions of the article in any way. The original article has been updated.

**Funding**

This work was supported by the Taipei Medical University Hospital (111TMUH-MOST-05) and the Ministry of Science and Technology of Taiwan (MOST 110-2628-B-038-014 and MOST 111-2628-B-038-022). The study was also supported by Taipei Medical University TMU108-AE1-B34 (PI. Yang Ching Chen).

