# Supplementary material for: Systolic blood pressure as the mediator of the effect of early menarche on the risk of coronary artery disease: A Mendelian randomization study
Source: Front Cardiovasc Med. 2023 Jan 9;9:1023355. doi: 10.3389/fcvm.2022.1023355 (PMC9868731; doi:10.3389/fcvm.2022.1023355)
Supplement: Supplementary file 1 [file Data_Sheet_1.pdf]

Supplementary Materials

Systolic Blood Pressure as the Mediator of the Effect of Early Menarche on the Risk of Coronary Artery Disease: A Mendelian Randomization Study

Hsien-Yu Fan, Yen-Tsung Huang, Yun-Yu Chen, Justin BoKai Hsu, Hung-Yuan Li, Ta-Chen Su, Hung-Ju Lin, Kuo-Liong Chien, Yang-Ching Chen

Supplementary methods 2

Chin-Shan Community Cardiovascular Cohort (CCCC) 2

Binary exposures and binary mediators 3

Mendelian randomization assumptions 3

Statistical analyses with binary exposures and binary mediators 4

R packages 7

Interactions 7

Inverse-odds-weighted estimation (IOW) 7

Sequential mediation results 8

Table S1. Genetic instruments 9

Table S2. Statistical packages used in the mediation analysis 10

Table S3. Mendelian randomization sensitivity analyses and external validation 11

Table S4. Summary results of single nucleotide polymorphisms on exposures and outcomes 13

Table S5. Summary results of single nucleotide polymorphisms on confounding factors 14

Table S6. P-values for interaction between the mediators and age at menarche or genetic risk scores of age at menarche on predicting risk of coronary artery diseases 15

Table S7. Effects of early menarche on the risk of coronary artery diseases using inverse-odds-weighted estimation (IOW) 16

Table S8. P-values for interaction between mediators and mediators on predicting risk of coronary artery diseases in Taiwan Biobank 17

Figure S1-1. Flowchart of participants in the Chin-Shan Community Cardiovascular Cohort and Taiwan Biobank surveys included in the present1 study 19

Figure S1-2. Flowchart for the statistical methods used in this analysis 20

Figure S2. Effects of early menarche on the risk of cardiovascular disease 21

Figure S3. Observational and genetically determined changes in mediators, associated with early menarche, translated into the risk of coronary artery diseases 22

Figure S4. Proportion of the mediated effects of early menarche on the risk of coronary artery disease 23

Figure S5. The pathway from age at menarche to the risk of coronary artery diseases (CAD) through body mass index (BMI) and systolic blood pressure (SBP) 24

Supplemental references 25

Supplementary methods

Chin-Shan Community Cardiovascular Cohort (CCCC)

The more information on the CCCC study is descripted in previous review as below:[1] *"The original idea of the CCCC study was unique. Professor Yuan-Teh Lee conducted this study, leading the faculty of the Division of Cardiology, Department of Internal Medicine, National Taiwan University, through the use of various facilitating resources, including theNational Science Council, Department of Health and the Foundation of Taiwan Electricity Company. This study began in 1990 by recruiting 1703 men and 1899 women at least 35 years of age and older, with homogeneous Chinese ethnicity, and living in the Chin-Shan township 30 km north of metropolitan Taipei, Taiwan. The participants' anthropometric and lifestyle data, medical history and current health conditions were assessed by interview questionnaires in 2-year cycles, and the validity and reproducibility of the collected data and measurements have been reported elsewhere in detail. Through the assistance of trained young faculty and medical students, we added to the original 3602 participants by collecting various questionnaires addressing basic lifestyle and behavioral information along with medical histories, clinical evaluation and physical examinations. At first, we measured the carotid artery intima media thickness and echocardiographic profiles in a systematic way. More recently, we further developed the bi-annual examination format to collect morbidity and mortality data according the following strategy: first, we surveyed the hospitalization and outpatient service records corresponding to the questionnaires; second, we regularly collected the death certificate documents from the local health station and performed oral interviews with relatives and family members about death events to confirm the causes of death."*

Binary exposures and binary mediators

Exposures

For the binary exposure variable, early menarche was defined as the lowest quartile of menarche timing based on individual data.[2] Therefore, in the Taiwan Biobank (TWB) study, early menarche was defined as menarche before the age of 13 years. Iin the Chin-Shan Community Cardiovascular Cohort (CCCC) study, early menarche was defined as menarche before the age of 14 years.

Mediators

Obesity, hypertension, dyslipidemia, and diabetes were included as binary mediator variables. A body mass index (BMI) over 27 kg・m^{-2} is considered obese. In the TWB study, participants were asked in a questionnaire whether a doctor had ever told them they had certain diseases such as hypertension, dyslipidemia, or diabetes. In the CCCC study, hypertension was diagnosed in individuals with systolic blood pressure at or above 140 mmHg and/or diastolic blood pressure at or above 90 mmHg, and in persons who were on antihypertension medications.[3] Diabetes mellitus was diagnosed in those with fasting blood sugar levels higher than 140 mg/dl and in those with oral hypoglycemic agents or insulin treatment.[3] Dyslipidemia was defined as a history of hyperlipidemia confirmed by cardiovasculogists.

Mendelian randomization assumptions

Tyler J. VanderWeele and their colleagues proposed Methodological challenges in Mendelian randomization and published this issue in Epidemiology in 2014.[4] According to this reference, three core MR assumptions are that *(1) the genetic marker is associated with the exposure, (2) the genetic marker is independent of the outcome given the exposure and all confounders (measured and unmeasured) of the exposure-outcome association, and (3) the genetic marker is independent of factors (measured and unmeasured) that confound the exposure-outcome relationship*.

Statistical analyses with binary exposures and binary mediators

We started with investigating the total effects on cardiovascular diseases including cerebrovascular accident and coronary artery diseases. Our three complementary approaches consistently indicated the total effect of early menarche on the risk of CAD (Figure S2). Therefore, we investigated the role of metabolic mediators in this effect on CAD outcomes (Figure S3). Finally, we found hypertension in mediating this effect (Figure S4).

Total effects of early menarche on the CVD outcomes (Figure S2)

In the MR analysis, the effects of menarche timing on CVD outcomes were investigated using two regression stages: the first stage involved the regression of early menarche on its genetic instruments. Subsequently, the predicted values of early menarche and residuals from the first stage were used in the second stage of logistic regression between CAD outcomes and the predicted value of early menarche. We adjusted all the analyses for the following confounders: age, education, current smoking, menopause, and regular physical activity.

In the TWB observational study, we performed logistic regression models to estimate the

association of early menarche with CVD outcomes. The models were used to estimate the odds ratios (ORs) and 95% confidence intervals (CIs). For each of the CVD outcomes, we fitted logistic regression models with age, education, current smoking, menopause, and regular physical activity.

In the CCCC study, we performed Cox proportional-hazards models to evaluate the effect of early menarche on the 15-year risk of CVD outcomes. The models were used to estimate the hazard ratios (HRs) and 95% confidence intervals (CIs). Before using Cox proportional-hazards models, we excluded 34 participants with cardiovascular diseases at baseline (Figure S1-1). For each of CVD outcomes, we fitted Cox models with age, education, current smoking, menopause, and regular physical activity.

Indirect effects of early menarche on binary mediators (Figure S3-A)

In the MR analysis, an effect of early menarche on each binary mediator variable was investigated using two regression stages: the first stage involved the regression of early menarche on its genetic instruments. Subsequently, the predicted values of early menarche and residuals from the first stage were used in the second stage of logistic regression between binary mediator variables and the predicted values of early menarche. The second stage models were adjusted for age, education, current smoking, menopause, and regular physical activity.

In TWB observational study and the CCCC study, we used logistic regression models to estimate the association of early menarche with binary mediator variables. The logistic regression models were adjusted for age, education, current smoking, menopause, and regular

physical activity.

Indirect effects of each mediator on the risk of CAD (Figure S3-B)

We estimated the effect of mediators on the risk of CAD, with additional adjustment for early menarche. In the MR analysis, an effect of a mediator variable on the CAD outcome was investigated using two regression stages: the first stage involved the regression of the mediator variable on its genetic instruments. Subsequently, the predicted values of the mediator and residuals from the first stage were used in the second stage of logistic regression between the CAD outcome and the predicted values of the mediator variable. The second stage models were adjusted for the predicted early menarche and confounders.

In the TWB observational study, we performed logistic regression models to estimate the association of mediators with the CAD outcome. All models were adjusted not only for confounding factors but also for early menarche.

In the CCCC study, we performed Cox proportional-hazards models to assess the association between mediators and the risk of CAD. After excluding participants with a history of CAD at baseline, we fitted Cox models with early menarche and confounding factors.

Proportion mediated (Figure S4)

In mediation analysis, there were two pathways by which the indirect effect may occur. The first pathway was from early menarche to mediators. The second pathway was from mediators to the CAD outcome. The two path coefficients were multiplied together to estimate the

indirect effect.[5,6] The proportion of the mediated effect was calculated by dividing the indirect effect by the total effect.[5,6] Standard errors were derived through bootstrapping in the two cohort analyses.[6] The proportion mediated is defined as "not causal" if the total and indirect effects have opposite directions.[7]

R packages

We used the package “mediation” to perform a causal mediation analysis in both CCCC and TWB studies. The following MR packages were applied to TWB study only (Table S2). The one-sample analysis was performed using two-stage regression models with the package “mediation” and “ivreg”. The sensitivity analyses of the one-sample MR were performed using the package “MendelianRandomization”.

Interactions

An assumption for mediation analysis is lack of interaction between age at menarche and mediators on the end point. We found an interaction between age at menarche and BMI in the MR association (Table S6; Bonferroni P = 0.007), and estimates from the MR mediation analysis for age at menarche should therefore be interpreted with some caution.

Inverse-odds-weighted estimation (IOW)

As Table S7, we have done this analysis and estimated three effects on the risk of CAD (Table R2): total effect (RR: 2.73; 95%CI: 1.24 to 5.88), natural direct effect (RR: 3.60; 95%CI: 1.24 to 8.20), and natural indirect effect (RR: 0.76; 95%CI: 0.46 to 1.36). This finding did not support our three main analyses. For this reason, this pathway may be confounded by other

factors. We suggested that future studies are warranted to use IOW to confirm this finding again.

Sequential mediation results

In this study design, we assumed that all mediators are parallel. Here, we have tried to use a sequential mediation approach[8] to test the pathway from age at menarche to the risk of coronary artery diseases (CAD) through body mass index (BMI) and systolic blood pressure (SBP). As below figure, we found two pathways (Figure S5):

Age at menarche ---> Body mass index ---> CAD risk

Age at menarche ---> Body mass index ---> Systolic blood pressure ---> CAD risk

Table S1. Genetic instruments

Phenotype	Number of SNPs	Pleiotropic effects (Excluded)	Number of instruments (Included)	N	F	Regression coefficient	SE	*P* value
**Continuous variable**								
Age at menarche	44	19	25	58,216	162.30	46.69	3.66	2.0E-16
BMI	32	14	18	58,212	41.14	35.79	5.58	1.4E-10
SBP	14	6	8	58,198	17.59	15.96	3.81	2.8E-05
DBP	14	6	8	58,198	12.27	7.65	2.18	4.6E-04
TC	7	3	4	58,214	243.20	8.05	0.52	2.0E-16
LDL	7	3	4	58,214	296.10	7.97	0.46	2.0E-16
HDL	7	3	4	58,214	312.60	8.07	0.46	2.0E-16
FBG	18	8	10	58,213	41.05	6.08	0.95	1.5E-10
**Dichotomous variables**								
Early menarche	44	19	25	58,216	137.70	64.93	5.55	2.0E-16
Obesity	32	14	18	58,213	20.68	18.59	4.09	5.5E-06
Hypertension	14	6	8	30,479	13.69	2.79	0.75	2.2E-04
Dyslipidemia	7	3	4	52,205	14.60	0.23	0.06	1.3E-04
Diabetes	18	8	10	38,397	38.83	37.88	6.09	5.0E-10

BMI: body mass index; DBP: diastolic blood pressure; FBG: fasting blood glucose; HDL: high-density lipoprotein cholesterol; LDL: low-density lipoprotein cholesterol; SE: standard error; SBP: systolic blood pressure; SNP: single-nucleotide polymorphism; N: number of participants; TC: total cholesterol.

Table S2. Statistical packages used in the mediation analysis

Method	Study	Package	Version	Published date
Conventional mediation analysis	Prospective cohort (CCCC)	"mediation"	4.5.0	2019-9-13
	Retrospective cohort (TWB)	"mediation"	4.5.0	2019-9-13
Mendelian randomization analysis	One-sample MR (TWB)	"ivreg" and "mediation"	0.6-1	2021-10-15
	MR sensitivity analysis (TWB)	"MendelianRandomization"	0.6.0	2022-02-22

CCCC: the Chin-Shan Community Cardiovascular Cohort study; MR: Mendelian randomization; TWB: the Taiwan Biobank study.

Table S3. Mendelian randomization sensitivity analyses and external validation

Pathway	Method	One-sample MR		
		Beta (95% CI)		*P*
Earlier age at menarche–CVD	IVW (random)	1.20	(0.22 to 2.18)	0.01
	IVW (fixed)	1.20	(0.22 to 2.18)	0.02
	MR-Egger	0.14	(−1.31 to 1.59)	0.85
	Simple median	2.63	(0.65 to 4.62)	0.01
	Weighted median	0.57	(−0.72 to 1.86)	0.39
	Maximum likelihood	1.16	(0.11 to 2.21)	0.03
Earlier age at menarche–CVA	IVW (random)	0.01	(−1.46 to 1.48)	0.99
	IVW (fixed)	0.01	(−1.46 to 1.48)	0.99
	MR-Egger	0.24	(−1.96 to 2.45)	0.82
	Simple median	−0.81	(−3.69 to 2.04)	0.57
	Weighted median	−0.45	(−2.48 to 1.58)	0.66
	Maximum likelihood	0.01	(−1.61 to 1.63)	0.99
Earlier age at menarche–CAD	IVW (random)	1.22	(0.08 to 2.37)	0.03
	IVW (fixed)	1.25	(0.11 to 2.39)	0.03
	MR-Egger	0.34	(−1.33 to 2.03)	0.68
	Simple median	1.02	(−1.19 to 3.23)	0.36
	Weighted median	0.62	(−0.89 to 2.13)	0.42
	Maximum likelihood	1.30	(0.09 to 2.50)	0.03
Earlier age at menarche–BMI	IVW (random)	0.26	(−0.15 to 0.68)	0.21
	IVW (fixed)	0.26	(−0.09 to 0.62)	0.15
	MR-Egger	0.24	(−0.33 to 0.81)	0.40
	Simple median	0.29	(−0.57 to 1.16)	0.51
	Weighted median	0.28	(−0.32 to 0.89)	0.36
	Maximum likelihood	0.26	(−0.20 to 0.72)	0.26
Earlier age at menarche–SBP	IVW (random)	0.38	(−2.25 to 3.01)	0.77
	IVW (fixed)	0.38	(−1.34 to 2.11)	0.66
	MR-Egger	−0.65	(−4.14 to 2.83)	0.71
	Simple median	2.33	(−2.52 to 7.18)	0.34
	Weighted median	0.30	(−2.57 to 3.19)	0.83
	Maximum likelihood	0.36	(−2.80 to 3.53)	0.82
Earlier age at menarche–DBP	IVW (random)	0.74	(−0.68 to 2.16)	0.30
	IVW (fixed)	0.74	(−0.25 to 1.73)	0.14
	MR-Egger	−0.18	(−2.07 to 1.70)	0.84
	Simple median	1.93	(−0.72 to 4.60)	0.15
	Weighted median	1.74	(0.05 to 3.44)	0.04
	Maximum likelihood	0.72	(−0.95 to 2.40)	0.39
Earlier age at menarche–TC	IVW (random)	−2.38	(−5.86 to 1.09)	0.17
	IVW (fixed)	−2.38	(−5.86 to 1.09)	0.17
	MR-Egger	−0.71	(−5.86 to 4.44)	0.78
	Simple median	−1.25	(−8.53 to 6.03)	0.73
	Weighted median	−1.11	(−6.25 to 4.02)	0.67
	Maximum likelihood	−2.50	(−6.27 to 1.26)	0.19
Earlier age at menarche–LDL	IVW (random)	−2.12	(−5.68 to 1.43)	0.24
	IVW (fixed)	−2.12	(−5.20 to 0.95)	0.17
	MR-Egger	−0.59	(−5.58 to 4.39)	0.81
	Simple median	−1.40	(−8.36 to 5.55)	0.69
	Weighted median	−0.96	(−5.62 to 3.69)	0.68
	Maximum likelihood	−2.40	(−6.30 to 1.50)	0.22
Earlier age at menarche–HDL	IVW (random)	−0.25	(−1.53 to 1.02)	0.69
	IVW (fixed)	−0.25	(−1.53 to 1.02)	0.69
	MR-Egger	−0.27	(−2.16 to 1.61)	0.77
	Simple median	0.72	(−1.82 to 3.27)	0.57
	Weighted median	−0.29	(−2.04 to 1.45)	0.74
	Maximum likelihood	−0.29	(−1.66 to 1.07)	0.67
Earlier age at menarche–FBG	IVW (random)	0.74	(−1.05 to 2.54)	0.41
	IVW (fixed)	0.74	(−1.05 to 2.54)	0.41
	MR-Egger	0.88	(−1.79 to 3.55)	0.51
	Simple median	−0.91	(−4.66 to 2.82)	0.63
	Weighted median	1.02	(−1.54 to 3.59)	0.43
	Maximum likelihood	0.71	(−1.21 to 2.64)	0.46
	IVW (random)	NA		0.99

BMI–CAD	IVW (fixed)	NA		0.99
	MR-Egger	NA		0.99
	Simple median	1.25	(−0.11 to 2.62)	0.07
	Weighted median	NA		0.99
	Maximum likelihood	NA		0.99
SBP–CAD	IVW (random)	0.32	(0.03 to 0.62)	0.02
	IVW (fixed)	0.32	(0.03 to 0.62)	0.02
	MR-Egger	0.26	(−0.17 to 0.70)	0.23
	Simple median	0.49	(−0.13 to 1.13)	0.12
	Weighted median	0.26	(−2.12 to 2.65)	0.82
	Maximum likelihood	0.41	(0.03 to 0.78)	0.03
DBP–CAD	IVW (random)	0.13	(−0.40 to 0.67)	0.62
	IVW (fixed)	0.13	(−0.21 to 0.58)	0.55
	MR-Egger	0.42	(−0.49 to 1.35)	0.36
	Simple median	−0.14	(−1.14 to 0.85)	0.77
	Weighted median	0.34	(−0.38 to 1.08)	0.35
	Maximum likelihood	0.26	(−0.53 to 1.06)	0.52
TC–CAD	IVW (random)	0.04	(−0.01 to 0.10)	0.17
	IVW (fixed)	0.04	(0.001 to 0.08)	0.04
	MR-Egger	−0.03	(−0.12 to 0.06)	0.50
	Simple median	0.09	(0.01 to 0.17)	0.03
	Weighted median	0.03	(−0.01 to 0.07)	0.18
	Maximum likelihood	0.04	(−0.01 to 0.11)	0.16
LDL–CAD	IVW (random)	0.06	(0.01 to 0.11)	0.01
	IVW (fixed)	0.06	(0.02 to 0.11)	0.01
	MR-Egger	0.03	(−0.04 to 0.10)	0.39
	Simple median	0.06	(−0.02 to 0.16)	0.14
	Weighted median	0.04	(−0.004 to 0.09)	0.07
	Maximum likelihood	0.06	(0.01 to 0.11)	0.01
HDL–CAD	IVW (random)	0.02	(−0.15 to 0.21)	0.76
	IVW (fixed)	0.02	(−0.07 to 0.12)	0.58
	MR-Egger	0.03	(−0.22 to 0.30)	0.77
	Simple median	0.58	(−2.58 to 3.75)	0.71
	Weighted median	0.02	(−0.27 to 0.33)	0.85
	Maximum likelihood	0.02	(−0.16 to 0.21)	0.76
FBG–CAD	IVW (random)	0.21	(0.08 to 0.34)	0.001
	IVW (fixed)	0.21	(0.08 to 0.34)	0.001
	MR-Egger	0.15	(−0.27 to 0.58)	0.47
	Simple median	0.24	(0.03 to 0.44)	0.01
	Weighted median	0.19	(0.02 to 0.35)	0.02
	Maximum likelihood	0.22	(0.08 to 0.35)	0.002

BMI: body mass index; CAD: coronary artery disease; CI: confidence interval; DBP: diastolic blood pressure; FBG: fasting blood glucose; HDL: high-density lipoprotein cholesterol; LDL: low-density lipoprotein cholesterol; SBP: systolic blood pressure; IVW: inverse variance weighted meta-analysis; ML: maximum-likelihood; NA: not applicable; TC: total cholesterol.

Table S4. Summary results of single nucleotide polymorphisms on exposures and outcomes

Traits	Abbreviation	SNP	CHR	Position	Minor allele	MAF	*P* values of menarche timing		*P* values of CVD outcomes			*P* values of continuous mediators							*P* values of binary mediators			
							AAM	EM	CVD	CVA	CAD	BMI	SBP	DBP	TC	LDL	HDL	FBG	Obesity	Hypertension	Dyslipidemia	Diabetes
Age at menarche	AAM	rs1038903	4	28750432	C	0.010	**0.599**	**0.980**	0.893	0.905	0.820	0.141	0.067	0.199	0.561	0.615	0.622	0.630	0.083	0.981	0.720	0.949
Age at menarche	AAM	rs10738889	9	32458055	G	0.410	**0.867**	**0.502**	0.139	0.707	0.124	0.040	0.620	0.952	0.240	0.215	0.888	0.091	0.005	0.440	0.286	0.527
Age at menarche	AAM	rs10789181	1	65350884	A	0.199	**0.147**	**0.076**	0.382	0.762	0.313	0.565	0.268	0.515	0.304	0.222	0.770	0.291	0.571	0.729	0.319	0.543
Age at menarche	AAM	rs10817638	9	114560262	G	0.416	**0.928**	**0.788**	0.853	0.731	0.976	0.303	0.053	0.073	0.863	0.934	0.932	0.841	0.432	0.729	0.444	0.700
Age at menarche	AAM	rs10846260	12	15971208	G	0.170	**0.059**	**0.066**	0.587	0.554	0.823	0.782	0.540	0.458	0.608	0.241	0.056	0.897	0.657	0.608	0.837	0.707
Age at menarche	AAM	rs17171818	5	138389314	C	0.407	**0.185**	**0.962**	0.067	0.585	0.014	0.731	0.590	0.730	0.125	0.008	0.099	0.925	0.892	0.254	0.523	0.527
Age at menarche	AAM	rs17268785	2	56364948	G	0.119	**0.016**	**0.000**	0.247	0.563	0.186	0.768	0.089	0.269	0.758	0.792	0.762	0.517	0.411	0.094	0.619	0.901
Age at menarche	AAM	rs2153127	6	104900669	T	0.440	**0.000**	**0.000**	0.237	0.129	0.550	0.395	0.077	0.179	0.027	0.014	0.569	0.377	0.767	0.164	0.424	0.887
Age at menarche	AAM	rs251130	5	111524099	A	0.245	**0.374**	**0.376**	0.013	0.025	0.214	0.320	0.005	0.005	0.361	0.476	0.935	0.495	0.424	0.022	0.725	0.449
Age at menarche	AAM	rs3101336	1	72285502	T	0.083	**0.373**	**0.191**	0.228	0.180	0.606	0.180	0.545	0.841	0.640	0.567	0.957	0.595	0.330	0.284	0.644	0.850
Age at menarche	AAM	rs3733631	4	103719946	C	0.334	**0.000**	**0.000**	0.481	0.603	0.284	0.276	0.013	0.025	0.819	0.430	0.697	0.941	0.049	0.781	0.243	0.449
Age at menarche	AAM	rs3893384	15	79680713	C	0.313	**0.745**	**0.610**	0.552	0.700	0.879	0.725	0.077	0.198	0.776	0.631	0.331	0.209	0.738	0.586	0.481	0.642
Age at menarche	AAM	rs466639	1	165425645	T	0.138	**0.000**	**0.000**	0.890	0.811	0.665	0.004	0.161	0.042	0.888	0.991	0.788	0.437	0.002	0.156	0.015	0.839
Age at menarche	AAM	rs4735738	8	76699390	G	0.447	**0.023**	**0.083**	0.024	0.021	0.466	0.171	0.181	0.980	0.010	0.024	0.073	0.186	0.617	0.896	0.012	0.047
Age at menarche	AAM	rs630602	1	54263191	C	0.305	**0.004**	**0.000**	0.779	0.954	0.796	0.365	0.197	0.041	0.089	0.046	0.113	0.459	0.114	0.097	0.654	0.564
Age at menarche	AAM	rs6427782	1	199829211	G	0.344	**0.006**	**0.065**	0.825	0.935	0.951	0.111	0.870	0.401	0.854	0.337	0.954	0.128	0.184	0.735	0.791	0.101
Age at menarche	AAM	rs6747380	2	56360614	A	0.118	**0.018**	**0.000**	0.259	0.549	0.193	0.791	0.104	0.301	0.764	0.769	0.812	0.502	0.437	0.091	0.636	0.904
Age at menarche	AAM	rs6762477	3	50055776	G	0.111	**0.416**	**0.177**	0.645	0.812	0.645	0.026	0.044	0.017	0.213	0.380	0.089	0.258	0.086	0.878	0.590	0.561
Age at menarche	AAM	rs6933660	6	151482619	C	0.205	**0.000**	**0.004**	0.735	0.888	0.732	0.358	0.970	0.402	0.562	0.757	0.547	0.531	0.217	0.290	0.761	0.444
Age at menarche	AAM	rs7701886	5	154167849	A	0.034	**0.508**	**0.702**	0.079	0.991	0.043	0.609	0.624	0.317	0.877	0.975	0.899	0.382	0.742	0.079	0.187	0.784
Age at menarche	AAM	rs8041943	15	79674258	G	0.297	**0.872**	**0.855**	0.629	0.661	0.755	0.619	0.094	0.248	0.889	0.905	0.298	0.329	0.981	0.459	0.634	0.313
Age at menarche	AAM	rs900145	11	13272358	C	0.454	**0.034**	**0.091**	0.732	0.436	0.792	0.451	0.056	0.148	0.476	0.458	0.941	0.122	0.801	0.088	0.723	0.265
Age at menarche	AAM	rs9475752	6	56915943	T	0.039	**0.957**	**0.591**	0.672	0.436	0.636	0.451	0.159	0.119	0.886	0.164	0.029	0.848	0.314	0.988	0.662	0.615
Age at menarche	AAM	rs9849248	3	88192124	C	0.179	**0.084**	**0.066**	0.392	0.785	0.545	0.758	0.228	0.031	0.936	0.839	0.499	0.446	0.577	0.530	0.726	0.090
Age at menarche	AAM	rs988913	6	54891510	C	0.121	**0.538**	**0.802**	0.711	0.375	0.847	0.559	0.477	0.943	0.198	0.335	0.575	0.094	0.425	0.127	0.770	0.177
Body mass index	BMI	rs11165643	1	96458541	C	0.195	0.101	0.137	0.595	0.766	0.456	**0.139**	0.317	0.179	0.020	0.053	0.612	0.265	**0.457**	0.384	0.331	0.473
Body mass index	BMI	rs11583200	1	50094148	T	0.083	0.346	0.699	0.123	0.780	0.110	**0.361**	0.044	0.279	0.159	0.194	0.016	0.678	**0.126**	0.146	0.454	0.711
Body mass index	BMI	rs12885454	14	29267632	C	0.492	0.060	0.670	0.815	0.183	0.257	**0.003**	0.810	0.374	0.920	0.484	0.140	0.342	**0.003**	0.164	0.008	0.812
Body mass index	BMI	rs12940622	17	80641771	A	0.303	0.973	0.396	0.064	0.294	0.055	**0.063**	0.475	0.650	0.945	0.790	0.835	0.690	**0.017**	0.753	0.735	0.993
Body mass index	BMI	rs13201877	6	137354404	G	0.039	0.214	0.124	0.232	0.284	0.460	**0.061**	0.820	0.937	0.338	0.314	0.635	0.488	**0.871**	0.771	0.676	0.245
Body mass index	BMI	rs2033732	8	84167474	T	0.403	0.366	0.082	0.081	0.007	0.610	**0.024**	0.295	0.091	0.341	0.594	0.332	0.383	**0.184**	0.114	0.118	0.048
Body mass index	BMI	rs205262	6	34595387	G	0.124	0.265	0.391	0.128	0.244	0.205	**0.987**	0.965	0.356	0.024	0.061	0.608	0.379	**0.657**	0.800	0.360	0.159
Body mass index	BMI	rs2365389	3	61250788	C	0.125	0.071	0.287	0.766	0.968	1.000	**0.049**	0.372	0.374	0.290	0.071	0.133	0.497	**0.037**	0.527	0.271	0.722
Body mass index	BMI	rs2820292	1	201815159	C	0.203	0.018	0.023	0.959	0.723	0.674	**0.421**	0.758	0.879	0.362	0.748	0.028	0.245	**0.057**	0.324	0.173	0.539
Body mass index	BMI	rs2836754	21	38919816	C	0.376	0.812	0.370	0.296	0.563	0.729	**0.035**	0.808	0.406	0.477	0.517	0.264	0.601	**0.354**	0.364	0.667	0.460
Body mass index	BMI	rs3101336	1	72285502	T	0.083	0.373	0.191	0.228	0.180	0.606	**0.180**	0.545	0.841	0.640	0.567	0.957	0.595	**0.330**	0.284	0.644	0.850
Body mass index	BMI	rs492400	2	218485029	C	0.235	0.009	0.017	0.306	0.366	0.642	**0.547**	0.274	0.404	0.893	0.813	0.840	0.652	**0.289**	0.497	0.801	0.466
Body mass index	BMI	rs6091540	20	52471323	T	0.294	0.174	0.416	0.394	0.112	0.097	**0.146**	0.041	0.046	0.558	0.955	0.005	0.187	**0.661**	0.818	0.492	0.453
Body mass index	BMI	rs6465468	7	95540202	T	0.014	0.542	0.244	0.980	0.885	0.514	**0.383**	0.958	0.268	0.699	0.947	0.383	0.837	**0.907**	0.450	0.882	0.857
Body mass index	BMI	rs7141420	14	79433111	T	0.468	0.403	0.550	0.448	0.782	0.602	**0.037**	0.743	0.587	0.183	0.021	0.027	0.280	**0.150**	0.595	0.949	0.186
Body mass index	BMI	rs7599312	2	212548507	A	0.023	0.450	0.066	0.619	0.059	0.353	**0.678**	0.047	0.136	0.584	0.222	0.021	0.351	**0.597**	0.847	0.339	0.631
Body mass index	BMI	rs7715256	5	154158333	T	0.035	0.521	0.725	0.097	0.967	0.059	**0.543**	0.678	0.374	0.801	0.972	0.879	0.438	**0.709**	0.093	0.263	0.923
Body mass index	BMI	rs9374842	6	119864519	C	0.088	0.029	0.104	0.329	0.162	0.999	**0.394**	0.072	0.015	0.318	0.400	0.522	0.606	**0.352**	0.062	0.184	0.623
Systolic blood pressure	SBP	rs805303	6	31648589	A	0.379	0.000	0.002	0.082	0.961	0.057	0.111	**0.379**	**0.088**	0.524	0.228	0.952	0.561	0.431	**0.085**	0.900	0.713
Systolic blood pressure	SBP	rs3184504	12	111446804	T	0.001	0.750	0.724	0.181	NA	0.081	0.882	**0.744**	**0.777**	0.887	0.322	0.777	0.289	0.993	**0.521**	0.953	0.964
Systolic blood pressure	SBP	rs6912283	6	43396756	G	0.308	0.437	0.694	0.018	0.036	0.088	0.779	**0.001**	**0.000**	0.104	0.040	0.211	0.799	0.926	**0.000**	0.379	0.891
Systolic blood pressure	SBP	rs11014166	10	18419869	T	0.144	0.102	0.070	0.359	0.361	0.658	0.979	**0.030**	**0.055**	0.211	0.573	0.096	0.488	0.873	**0.384**	0.382	0.208
Systolic blood pressure	SBP	rs6504640	17	46989720	T	0.395	0.630	0.675	0.936	0.962	0.924	0.774	**0.913**	**0.840**	0.081	0.460	0.028	0.264	0.554	**0.064**	0.258	0.250
Systolic blood pressure	SBP	rs17367504	1	11802721	G	0.124	0.703	0.762	0.362	0.962	0.376	0.519	**0.696**	**0.960**	0.112	0.129	0.013	0.110	0.568	**0.281**	0.031	0.667
Systolic blood pressure	SBP	rs2384550	12	114914926	A	0.175	0.313	0.234	0.169	0.045	0.840	0.162	**0.416**	**0.411**	0.911	0.828	0.711	0.373	0.322	**0.732**	0.701	0.754
Systolic blood pressure	SBP	rs16851412	3	141421817	A	0.181	0.534	0.536	0.987	0.791	0.987	0.142	**0.490**	**0.112**	0.505	0.399	0.413	0.229	0.954	**0.178**	0.841	0.546
Diastolic blood pressure	DBP	rs805303	6	31648589	A	0.379	0.000	0.002	0.082	0.961	0.057	0.111	**0.379**	**0.088**	0.524	0.228	0.952	0.561	0.431	**0.085**	0.900	0.713
Diastolic blood pressure	DBP	rs3184504	12	111446804	T	0.001	0.750	0.724	0.181	NA	0.081	0.882	**0.744**	**0.777**	0.887	0.322	0.777	0.289	0.993	**0.521**	0.953	0.964
Diastolic blood pressure	DBP	rs6912283	6	43396756	G	0.308	0.437	0.694	0.018	0.036	0.088	0.779	**0.001**	**0.000**	0.104	0.040	0.211	0.799	0.926	**0.000**	0.379	0.891
Diastolic blood pressure	DBP	rs11014166	10	18419869	T	0.144	0.102	0.070	0.359	0.361	0.658	0.979	**0.030**	**0.055**	0.211	0.573	0.096	0.488	0.873	**0.384**	0.382	0.208
Diastolic blood pressure	DBP	rs6504640	17	46989720	T	0.395	0.630	0.675	0.936	0.962	0.924	0.774	**0.913**	**0.840**	0.081	0.460	0.028	0.264	0.554	**0.064**	0.258	0.250
Diastolic blood pressure	DBP	rs17367504	1	11802721	G	0.124	0.703	0.762	0.362	0.962	0.376	0.519	**0.696**	**0.960**	0.112	0.129	0.013	0.110	0.568	**0.281**	0.031	0.667
Diastolic blood pressure	DBP	rs2384550	12	114914926	A	0.175	0.313	0.234	0.169	0.045	0.840	0.162	**0.416**	**0.411**	0.911	0.828	0.711	0.373	0.322	**0.732**	0.701	0.754
Diastolic blood pressure	DBP	rs16851412	3	141421817	A	0.181	0.534	0.536	0.987	0.791	0.987	0.142	**0.490**	**0.112**	0.505	0.399	0.413	0.229	0.954	**0.178**	0.841	0.546
Total cholesterol	TC	rs7525649	1	55033483	C	0.347	0.040	0.100	0.034	0.948	0.009	0.839	0.375	0.079	**0.000**	**0.000**	**0.841**	0.802	0.227	0.497	**0.850**	0.712
Total cholesterol	TC	rs157582	19	44892962	T	0.174	0.500	0.793	0.340	0.658	0.092	0.294	0.757	0.399	**0.000**	**0.000**	**0.148**	0.224	0.590	0.927	**0.964**	0.042
Total cholesterol	TC	rs2575876	9	104903458	A	0.230	0.747	0.659	0.515	0.452	0.655	0.915	0.065	0.188	**0.000**	**0.000**	**0.000**	0.810	0.229	0.902	**0.000**	0.472
Total cholesterol	TC	rs12453914	17	69142737	A	0.477	0.341	0.909	0.428	0.870	0.397	0.701	0.814	0.170	**0.000**	**0.000**	**0.777**	0.469	0.843	0.703	**0.082**	0.475
Low density lipoprotein	LDL	rs7525649	1	55033483	C	0.347	0.040	0.100	0.034	0.948	0.009	0.839	0.375	0.079	**0.000**	**0.000**	**0.841**	0.802	0.227	0.497	**0.850**	0.712
Low density lipoprotein	LDL	rs157582	19	44892962	T	0.174	0.500	0.793	0.340	0.658	0.092	0.294	0.757	0.399	**0.000**	**0.000**	**0.148**	0.224	0.590	0.927	**0.964**	0.042
Low density lipoprotein	LDL	rs2575876	9	104903458	A	0.230	0.747	0.659	0.515	0.452	0.655	0.915	0.065	0.188	**0.000**	**0.000**	**0.000**	0.810	0.229	0.902	**0.000**	0.472
Low density lipoprotein	LDL	rs12453914	17	69142737	A	0.477	0.341	0.909	0.428	0.870	0.397	0.701	0.814	0.170	**0.000**	**0.000**	**0.777**	0.469	0.843	0.703	**0.082**	0.475
High density lipoprotein	HDL	rs7525649	1	55033483	C	0.347	0.040	0.100	0.034	0.948	0.009	0.839	0.375	0.079	**0.000**	**0.000**	**0.841**	0.802	0.227	0.497	**0.850**	0.712
High density lipoprotein	HDL	rs157582	19	44892962	T	0.174	0.500	0.793	0.340	0.658	0.092	0.294	0.757	0.399	**0.000**	**0.000**	**0.148**	0.224	0.590	0.927	**0.964**	0.042
High density lipoprotein	HDL	rs2575876	9	104903458	A	0.230	0.747	0.659	0.515	0.452	0.655	0.915	0.065	0.188	**0.000**	**0.000**	**0.000**	0.810	0.229	0.902	**0.000**	0.472
High density lipoprotein	HDL	rs12453914	17	69142737	A	0.477	0.341	0.909	0.428	0.870	0.397	0.701	0.814	0.170	**0.000**	**0.000**	**0.777**	0.469	0.843	0.703	**0.082**	0.475
Fasting blood glucose	FBG	rs7578597	2	43505684	C	0.005	0.960	0.496	0.165	0.345	0.445	0.731	0.007	0.153	0.555	0.576	0.561	**0.427**	0.539	0.773	0.821	**0.204**
Fasting blood glucose	FBG	rs10010131	4	6291188	A	0.069	0.382	0.696	0.963	0.441	0.260	0.713	0.753	0.728	0.672	0.702	0.876	**0.003**	0.315	0.226	0.202	**0.003**
Fasting blood glucose	FBG	rs734312	4	6301627	G	0.171	0.363	0.510	0.814	0.726	0.371	0.254	0.170	0.861	0.787	0.964	0.444	**0.050**	0.101	0.836	0.676	**0.086**
Fasting blood glucose	FBG	rs17584499	9	8879118	T	0.092	0.523	0.612	0.253	0.596	0.436	0.382	0.730	0.243	0.015	0.020	0.949	**0.203**	0.503	0.108	0.239	**0.717**
Fasting blood glucose	FBG	rs5015480	10	92705802	C	0.187	0.841	0.511	0.676	0.820	0.881	0.029	0.021	0.110	0.010	0.009	0.231	**0.128**	0.014	0.053	0.156	**0.023**
Fasting blood glucose	FBG	rs1470579	3	185811292	C	0.250	0.623	0.331	0.466	0.847	0.185	0.161	0.031	0.036	0.954	0.965	0.780	**0.000**	0.139	0.159	0.611	**0.000**
Fasting blood glucose	FBG	rs6769511	3	185812502	C	0.250	0.630	0.344	0.464	0.848	0.184	0.165	0.032	0.037	0.967	0.986	0.811	**0.000**	0.140	0.159	0.572	**0.000**
Fasting blood glucose	FBG	rs4402960	3	185793899	T	0.240	0.865	0.345	0.320	0.976	0.133	0.250	0.020	0.014	0.942	0.955	0.963	**0.000**	0.154	0.060	0.388	**0.000**
Fasting blood glucose	FBG	rs4376068	3	185779847	C	0.239	0.812	0.302	0.362	0.867	0.202	0.276	0.020	0.011	0.932	0.894	0.822	**0.000**	0.146	0.071	0.373	**0.000**
Fasting blood glucose	FBG	rs4506565	10	112996282	T	0.023	0.696	0.250	0.105	0.546	0.134	0.756	0.514	0.796	0.706	0.758	0.074	**0.411**	0.510	0.241	0.761	**0.012**

AAM: age at menarche; BMI: body mass index; CAD: coronary artery disease; CHR: chromosome; CVA: cerebrovascular accident; CVD: cardiovascular disease; DBP: diastolic blood pressure; EM: early menarche; FBG: fasting blood glucose; HDL: high-density lipoprotein cholesterol; LDL: low-density lipoprotein cholesterol; MAF: minor allele frequency; SBP: systolic blood pressure; SNP: single nucleotide polymorphism; TC: total cholesterol

Table S5. Summary results of single nucleotide polymorphisms on confounding factors

Traits	Abbreviation	SNP	CHR	Position	Minor allele	MAF	*P* values of confounders				
							Age	Education	Physical activity	Smoking	Menopause
Age at menarche	AAM	rs1038903	4	28750432	C	0.010	0.218	0.307	0.662	0.099	0.117
Age at menarche	AAM	rs10738889	9	32458055	G	0.410	0.626	0.952	0.026	0.361	0.190
Age at menarche	AAM	rs10789181	1	65350884	A	0.199	0.471	0.886	0.846	0.615	0.374
Age at menarche	AAM	rs10817638	9	114560262	G	0.416	0.297	0.554	0.389	0.813	0.987
Age at menarche	AAM	rs10846260	12	15971208	G	0.170	0.396	0.390	0.073	0.555	0.297
Age at menarche	AAM	rs17171818	5	138389314	C	0.407	0.161	0.283	0.746	0.655	0.201
Age at menarche	AAM	rs17268785	2	56364948	G	0.119	0.976	0.364	0.460	0.845	0.519
Age at menarche	AAM	rs2153127	6	104900669	T	0.440	0.005	0.040	0.380	0.201	0.069
Age at menarche	AAM	rs251130	5	111524099	A	0.245	0.012	0.977	0.460	0.021	0.579
Age at menarche	AAM	rs3101336	1	72285502	T	0.083	0.508	0.564	0.540	0.878	0.576
Age at menarche	AAM	rs3733631	4	103719946	C	0.334	0.298	0.494	0.381	0.993	0.600
Age at menarche	AAM	rs3893384	15	79680713	C	0.313	0.340	0.236	0.477	0.480	0.258
Age at menarche	AAM	rs466639	1	165425645	T	0.138	0.082	0.331	0.002	0.153	0.013
Age at menarche	AAM	rs4735738	8	76699390	G	0.447	0.441	0.644	0.746	0.597	0.846
Age at menarche	AAM	rs630602	1	54263191	C	0.305	0.811	0.187	0.345	0.242	0.948
Age at menarche	AAM	rs6427782	1	199829211	G	0.344	0.825	0.081	0.259	0.918	0.101
Age at menarche	AAM	rs6747380	2	56360614	A	0.118	0.970	0.361	0.445	0.868	0.493
Age at menarche	AAM	rs6762477	3	50055776	G	0.111	0.323	0.565	0.909	0.743	0.339
Age at menarche	AAM	rs6933660	6	151482619	C	0.205	0.039	0.083	0.643	0.022	0.060
Age at menarche	AAM	rs7701886	5	154167849	A	0.034	0.900	0.565	0.365	0.798	0.639
Age at menarche	AAM	rs8041943	15	79674258	G	0.297	0.481	0.515	0.507	0.319	0.502
Age at menarche	AAM	rs900145	11	13272358	C	0.454	0.529	0.283	0.417	0.080	0.909
Age at menarche	AAM	rs9475752	6	56915943	T	0.039	0.603	0.920	0.574	0.908	0.385
Age at menarche	AAM	rs9849248	3	88192124	C	0.179	0.314	0.399	0.898	0.166	0.647
Age at menarche	AAM	rs988913	6	54891510	C	0.121	0.548	0.824	0.989	0.874	0.589
Body mass index	BMI	rs11165643	1	96458541	C	0.195	0.054	0.672	0.454	0.919	0.352
Body mass index	BMI	rs11583200	1	50094148	T	0.083	0.187	0.084	0.943	0.427	0.500
Body mass index	BMI	rs12885454	14	29267632	C	0.492	0.237	0.498	0.987	0.402	0.285
Body mass index	BMI	rs12940622	17	80641771	A	0.303	0.652	0.133	0.415	0.752	0.136
Body mass index	BMI	rs13201877	6	137354404	G	0.039	0.315	0.756	0.017	0.876	0.353
Body mass index	BMI	rs2033732	8	84167474	T	0.403	0.559	0.808	0.329	0.410	0.123
Body mass index	BMI	rs205262	6	34595387	G	0.124	0.977	0.769	0.881	0.837	0.924
Body mass index	BMI	rs2365389	3	61250788	C	0.125	0.524	0.688	0.012	0.157	0.437
Body mass index	BMI	rs2820292	1	201815159	C	0.203	0.636	0.189	0.465	0.351	0.123
Body mass index	BMI	rs2836754	21	38919816	C	0.376	0.233	0.324	0.021	0.254	0.143
Body mass index	BMI	rs3101336	1	72285502	T	0.083	0.508	0.564	0.540	0.878	0.576
Body mass index	BMI	rs492400	2	218485029	C	0.235	0.613	0.509	0.098	0.748	0.964
Body mass index	BMI	rs6091540	20	52471323	T	0.294	0.482	0.596	0.648	0.229	0.310
Body mass index	BMI	rs6465468	7	95540202	T	0.014	0.862	0.657	0.256	0.654	0.597
Body mass index	BMI	rs7141420	14	79433111	T	0.468	0.079	0.901	0.366	0.309	0.205
Body mass index	BMI	rs7599312	2	212548507	A	0.023	0.361	0.308	0.783	0.444	0.769
Body mass index	BMI	rs7715256	5	154158333	T	0.035	0.880	0.749	0.448	0.904	0.663
Body mass index	BMI	rs9374842	6	119864519	C	0.088	0.086	0.082	0.630	0.541	0.075
Systolic blood pressure	SBP	rs805303	6	31648589	A	0.379	0.522	0.817	0.689	0.159	0.229
Systolic blood pressure	SBP	rs3184504	12	111446804	T	0.001	0.246	0.156	0.588	0.720	0.861
Systolic blood pressure	SBP	rs6912283	6	43396756	G	0.308	0.904	0.209	0.438	0.642	0.165
Systolic blood pressure	SBP	rs11014166	10	18419869	T	0.144	0.021	0.681	0.234	0.372	0.354
Systolic blood pressure	SBP	rs6504640	17	46989720	T	0.395	0.911	0.633	0.337	0.552	0.240
Systolic blood pressure	SBP	rs17367504	1	11802721	G	0.124	0.070	0.525	0.470	0.010	0.674
Systolic blood pressure	SBP	rs2384550	12	114914926	A	0.175	0.662	0.012	0.166	0.164	0.893
Systolic blood pressure	SBP	rs16851412	3	141421817	A	0.181	0.714	0.725	0.905	0.427	0.912
Diastolic blood pressure	DBP	rs805303	6	31648589	A	0.379	0.522	0.817	0.689	0.159	0.229
Diastolic blood pressure	DBP	rs3184504	12	111446804	T	0.001	0.246	0.156	0.588	0.720	0.861
Diastolic blood pressure	DBP	rs6912283	6	43396756	G	0.308	0.904	0.209	0.438	0.642	0.165
Diastolic blood pressure	DBP	rs11014166	10	18419869	T	0.144	0.021	0.681	0.234	0.372	0.354
Diastolic blood pressure	DBP	rs6504640	17	46989720	T	0.395	0.911	0.633	0.337	0.552	0.240
Diastolic blood pressure	DBP	rs17367504	1	11802721	G	0.124	0.070	0.525	0.470	0.010	0.674
Diastolic blood pressure	DBP	rs2384550	12	114914926	A	0.175	0.662	0.012	0.166	0.164	0.893
Diastolic blood pressure	DBP	rs16851412	3	141421817	A	0.181	0.714	0.725	0.905	0.427	0.912
Total cholesterol	TC	rs7525649	1	55033483	C	0.347	0.485	0.872	0.647	0.892	0.927
Total cholesterol	TC	rs157582	19	44892962	T	0.174	0.201	0.986	0.258	0.757	0.134
Total cholesterol	TC	rs2575876	9	104903458	A	0.230	0.377	0.553	0.815	0.554	0.813
Total cholesterol	TC	rs12453914	17	69142737	A	0.477	0.447	0.040	0.065	0.902	0.972
Low density lipoprotein	LDL	rs7525649	1	55033483	C	0.347	0.485	0.872	0.647	0.892	0.927
Low density lipoprotein	LDL	rs157582	19	44892962	T	0.174	0.201	0.986	0.258	0.757	0.134
Low density lipoprotein	LDL	rs2575876	9	104903458	A	0.230	0.377	0.553	0.815	0.554	0.813
Low density lipoprotein	LDL	rs12453914	17	69142737	A	0.477	0.447	0.040	0.065	0.902	0.972
High density lipoprotein	HDL	rs7525649	1	55033483	C	0.347	0.485	0.872	0.647	0.892	0.927
High density lipoprotein	HDL	rs157582	19	44892962	T	0.174	0.201	0.986	0.258	0.757	0.134
High density lipoprotein	HDL	rs2575876	9	104903458	A	0.230	0.377	0.553	0.815	0.554	0.813
High density lipoprotein	HDL	rs12453914	17	69142737	A	0.477	0.447	0.040	0.065	0.902	0.972
Fasting blood glucose	FBG	rs7578597	2	43505684	C	0.005	0.876	0.131	0.893	0.718	0.447
Fasting blood glucose	FBG	rs10010131	4	6291188	A	0.069	0.185	0.971	0.113	0.377	0.287
Fasting blood glucose	FBG	rs734312	4	6301627	G	0.171	0.149	0.927	0.597	0.868	0.753
Fasting blood glucose	FBG	rs17584499	9	8879118	T	0.092	0.589	0.129	0.988	0.710	0.580
Fasting blood glucose	FBG	rs5015480	10	92705802	C	0.187	0.312	0.672	0.884	0.933	0.835
Fasting blood glucose	FBG	rs1470579	3	185811292	C	0.250	0.018	0.287	0.821	0.957	0.123
Fasting blood glucose	FBG	rs6769511	3	185812502	C	0.250	0.017	0.299	0.838	0.958	0.124
Fasting blood glucose	FBG	rs4402960	3	185793899	T	0.240	0.027	0.125	0.821	0.974	0.093
Fasting blood glucose	FBG	rs4376068	3	185779847	C	0.239	0.035	0.113	0.924	0.982	0.129
Fasting blood glucose	FBG	rs4506565	10	112996282	T	0.023	0.304	0.263	0.234	0.698	0.678

AAM: age at menarche; BMI: body mass index; CHR: chromosome; DBP: diastolic blood pressure; FBG: fasting blood glucose; HDL: high-density lipoprotein cholesterol; LDL: low-density lipoprotein cholesterol; MAF: minor allele frequency; SBP: systolic blood pressure; SNP: single nucleotide polymorphism; TC: total cholesterol

Table S6. P-values for interaction between the mediators and age at menarche or genetic risk scores of age at menarche on predicting risk of coronary artery diseases

The Mediators	P-value for interaction			
	Age at menarche		Genetic risk scores of age at menarche	
	P-value for interaction	P-value for interaction corrected for multiple comparison (Bonferroni)	P-value for interaction	P-value for interaction corrected for multiple comparison (Bonferroni)
BMI	0.063	0.378	0.001	0.007
SBP	0.416	1.00	0.017	0.085
DBP	0.843	0.843	0.062	0.248
TC	0.341	1.00	0.647	0.647
LDL	0.665	1.00	0.589	1.00
HDL	0.021	0.147	0.088	0.264
FBG	0.424	1.00	0.013	0.078

BMI: body mass index; CAD: coronary artery disease; DBP: diastolic blood pressure; FBG: fasting blood glucose; HDL: high-density lipoprotein cholesterol; LDL: low-density lipoprotein cholesterol; SBP: systolic blood pressure; TC: total cholesterol.

Table S7. Effects of early menarche on the risk of coronary artery diseases using inverse-odds-weighted estimation (IOW)

	RR	95%CI	
Total effect	2.73	1.24	5.88
Natural direct effect	3.60	1.24	8.20
Natural indirect effect	0.76	0.46	1.36

Table S8. P-values for interaction between mediators and mediators on predicting risk of coronary artery diseases in Taiwan Biobank

The Mediators	P-value for interaction					
	CCCC		Mediator		Genetic risk scores of mediators	
	P-value for interaction	P-value for interaction corrected for multiple comparison (Bonferroni)	P-value for interaction	P-value for interaction corrected for multiple comparison (Bonferroni)	P-value for interaction	P-value for interaction corrected for multiple comparison (Bonferroni)
BMI-SBP	0.057	0.912	0.044	0.31	0.369	1.00
BMI-DBP	0.007	0.147	0.067	0.40	0.499	1.00
BMI-TC	0.481	1.00	0.072	0.22	0.066	1.00
BMI-LDL	0.293	1.00	0.001	0.01	0.021	0.819
BMI-HDL	0.318	1.00	0.004	0.04	0.093	1.00
BMI-FBG	0.162	1.00	0.0001	1.6E-03	0.166	1.00
SBP-BMI	0.057	0.912	0.044	0.31	0.089	1.00
SBP-DBP	0.977	0.977	0.003	0.03	0.069	1.00
SBP-TC	0.016	0.304	0.0003	4.5E-03	0.719	1.00
SBP-LDL	0.010	0.20	3.41e-06	6.5E-05	0.423	1.00
SBP-HDL	0.278	1.00	0.007	0.06	0.155	1.00
SBP-FBG	0.081	1.00	0.071	0.28	0.943	0.943
DBP-BMI	0.007	0.147	0.067	0.40	0.806	1.00
DBP-SBP	0.977	0.977	0.003	0.03	0.091	1.00
DBP-TC	0.162	1.00	0.0028	0.03	0.277	1.00
DBP-LDL	0.035	0.595	0.0029	0.03	0.151	1.00
DBP-HDL	0.032	0.576	3.41e-06	6.8E-05	0.674	1.00
DBP -FBG	0.171	1.00	0.071	0.36	0.028	1.00
TC-BMI	0.481	1.00	0.072	0.22	0.238	1.00
TC-SBP	0.016	0.304	0.0003	4.5E-03	0.570	1.00
TC-DBP	0.016	0.304	0.0003	4.5E-03	0.597	1.00
TC-LDL	0.805	1.00	3.24e-05	5.5E-04	0.749	1.00
TC-HDL	0.167	1.00	0.298	0.60	0.234	1.00
TC-FBG	0.937	1.00	3.50e-06	6.3E-05	0.640	1.00
LDL-BMI	0.293	1.00	0.001	0.01	0.02	0.8

LDL-SBP	0.010	0.20	3.41e-06	6.5E-05	0.91	1.00
LDL-DBP	0.035	0.595	0.0029	0.03	0.51	1.00
LDL-TC	0.805	1.00	3.24e-05	5.5E-04	0.15	1.00
LDL-HDL	0.181	1.00	0.53	0.53	0.18	1.00
LDL-FBG	0.899	1.00	3.62e-09	7.6E-08	0.05	1.00
HDL-BMI	0.318	1.00	0.004	0.04	0.89	1.00
HDL-SBP	0.278	1.00	0.007	0.06	0.55	1.00
HDL-DBP	0.032	0.576	3.41e-06	6.8E-05	0.34	1.00
HDL-TC	0.167	1.00	0.298	0.60	0.78	1.00
HDL-LDL	0.181	1.00	0.53	0.53	0.82	1.00
HDL-FBG	0.231	1.00	0.0007	0.01	0.02	0.82
FBG-BMI	0.162	1.00	0.0001	1.6E-03	0.62	1.00
FBG-SBP	0.081	1.00	0.071	0.28	0.18	1.00
FBG-DBP	0.171	1.00	0.071	0.36	0.45	1.00
FBG-TC	0.937	1.00	3.50e-06	6.3E-05	0.11	1.00
FBG-LDL	0.899	1.00	3.62e-09	7.6E-08	0.007	0.294
FBG-HDL	0.231	1.00	0.0007	0.01	0.351	1.00

BMI: body mass index; CAD: coronary artery disease; DBP: diastolic blood pressure; FBG: fasting blood glucose; HDL: high-density lipoprotein cholesterol; LDL: low-density lipoprotein cholesterol; SBP: systolic blood pressure; TC: total cholesterol.

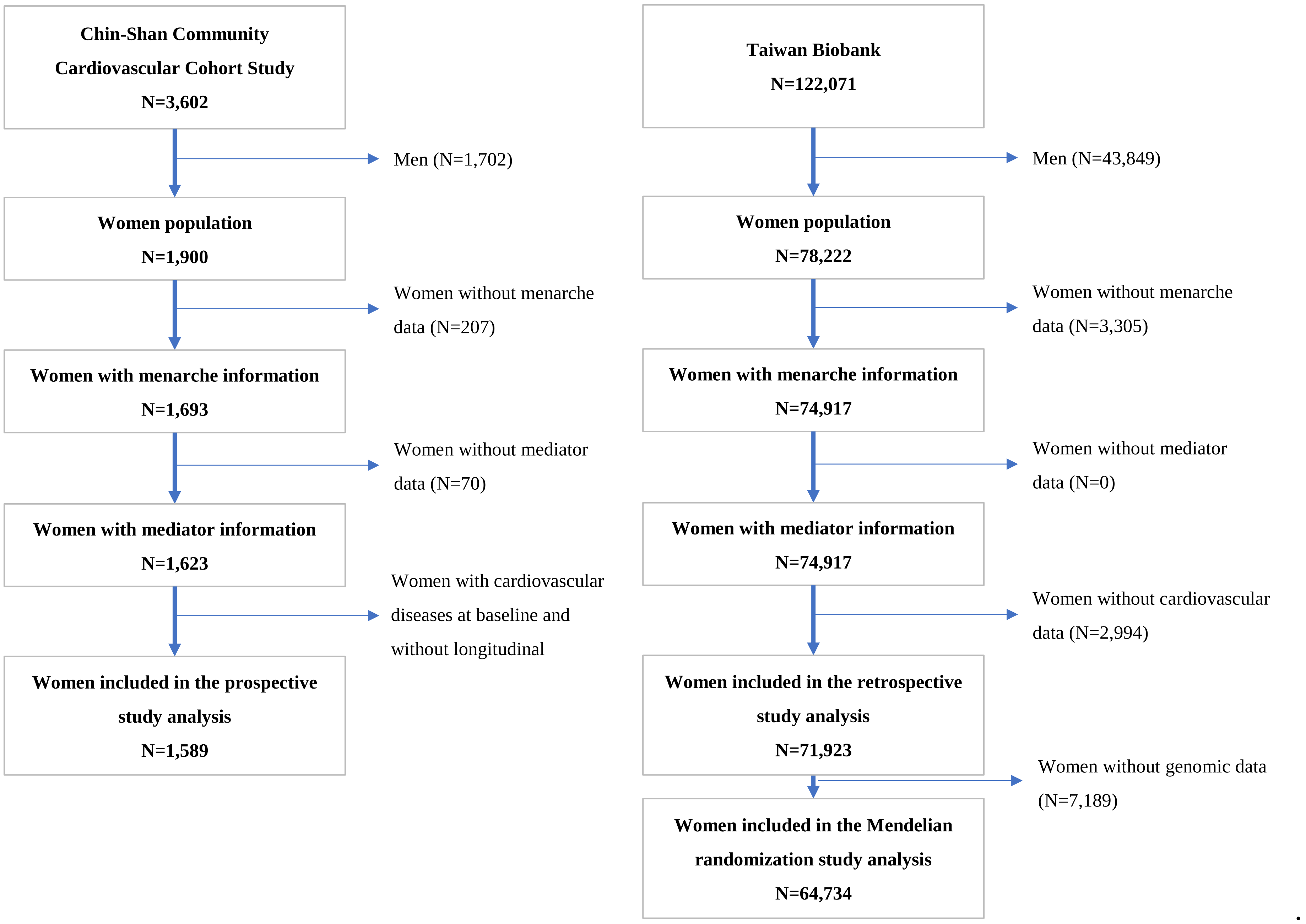

Figure S1-1. Flowchart of participants in the Chin-Shan Community Cardiovascular Cohort and Taiwan Biobank surveys included in the present1 study

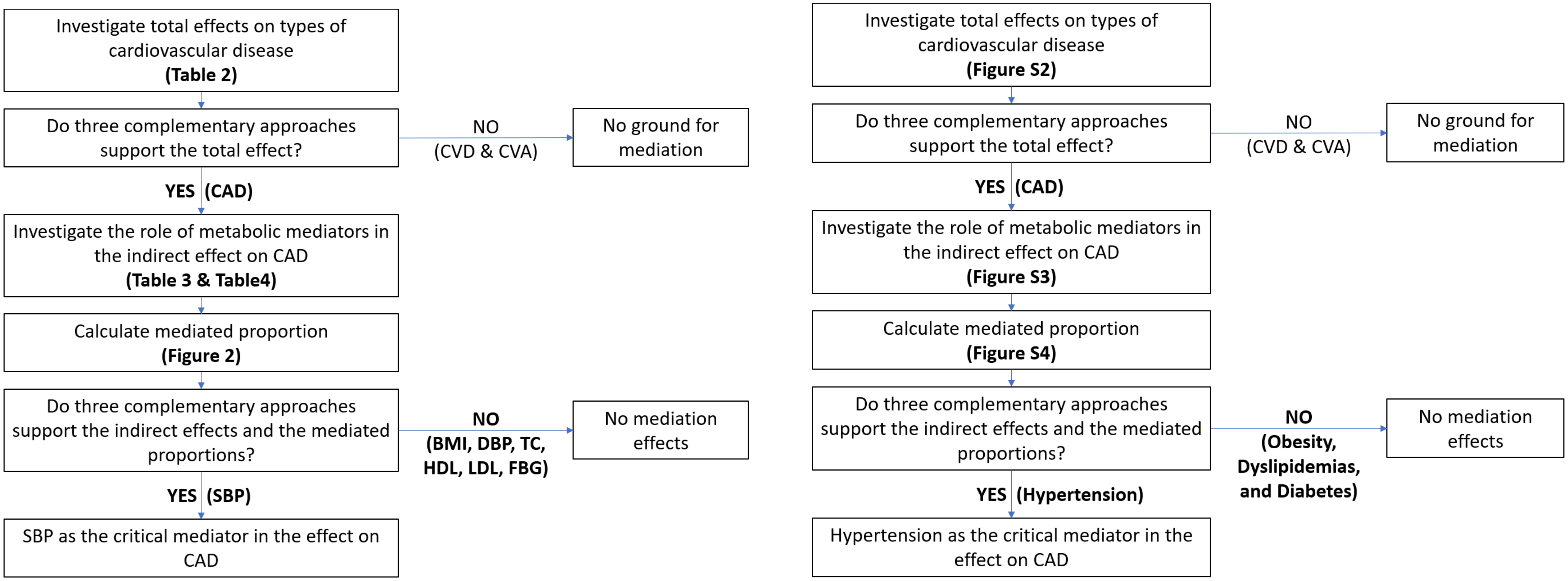

(A) The continuous exposure variable with continuous mediator variables

(B) The binary exposure variable with binary mediator variables

Figure S1-2. Flowchart for the statistical methods used in this analysis

Figure legend. BMI: body mass index; CAD: coronary artery diseases; CVA: cerebrovascular accident; CVD: cardiovascular disease; DBP: diastolic blood pressure; FBG: fasting blood glucose; HDL: high-density lipoprotein cholesterol; LDL: low-density lipoprotein cholesterol; SBP: systolic blood pressure; TC: total cholesterol.

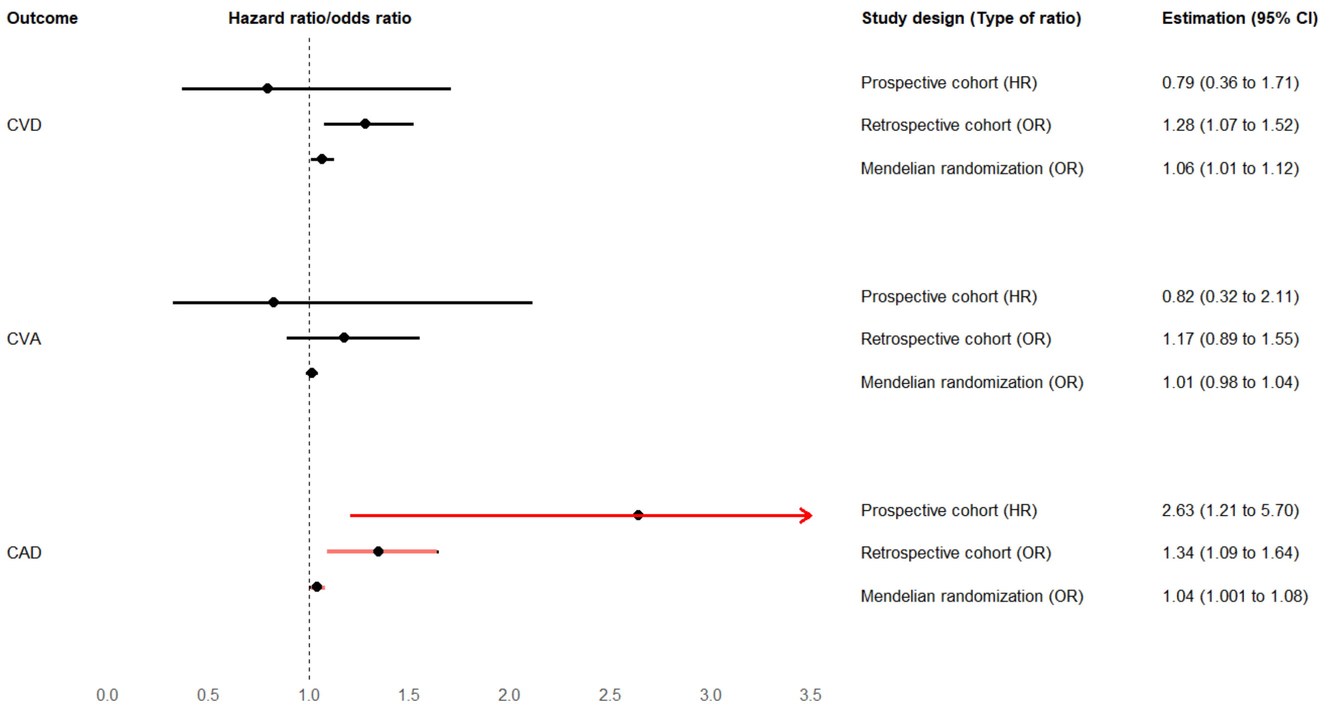

Figure S2. Effects of early menarche on the risk of cardiovascular disease

Figure legend. CAD: coronary artery diseases; CI: confidence interval; CVA: cerebrovascular accident; CVD: cardiovascular disease; HR: hazard ratio; OR: odds ratio.

(A)

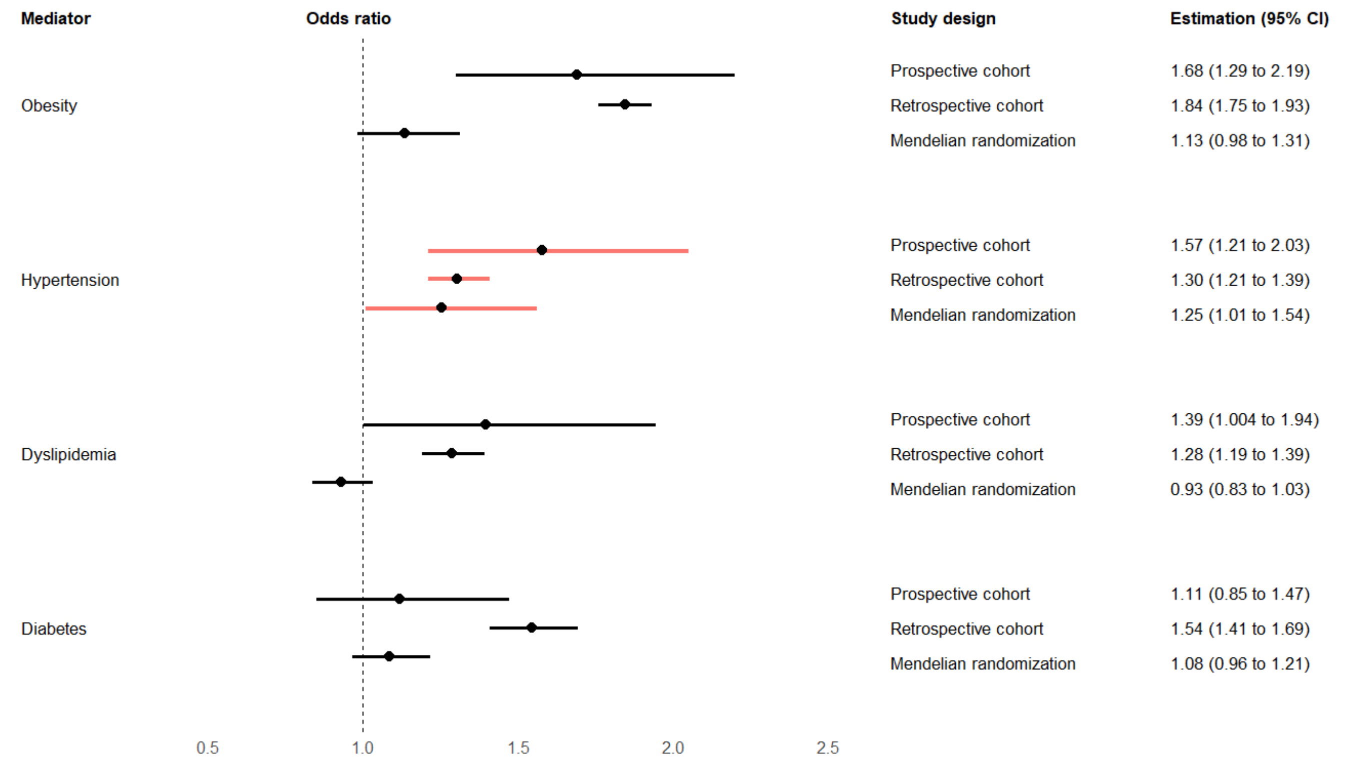

(B)

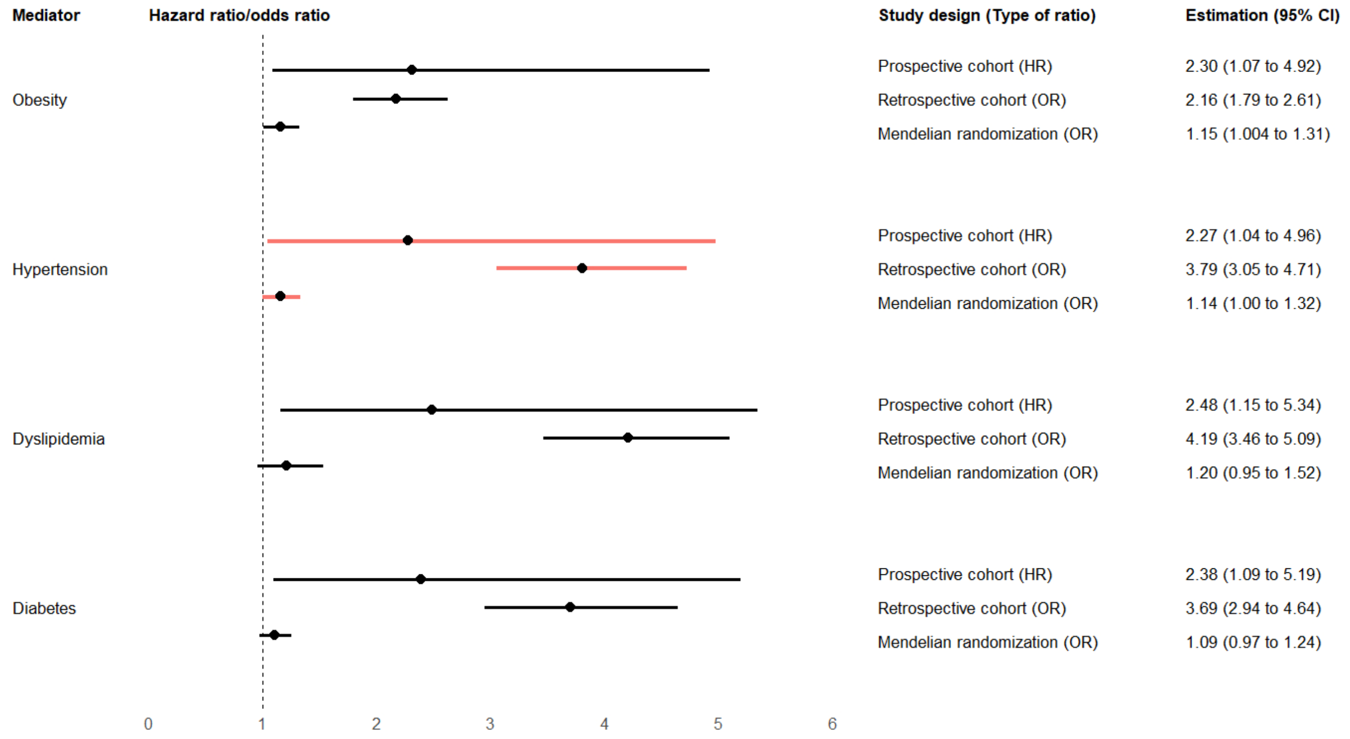

Figure S3. Observational and genetically determined changes in mediators, associated with early menarche, translated into the risk of coronary artery diseases

Figure legend. (A) Effects of early menarche on the risk of mediators; (B) Effects of mediators on the risk of coronary artery disease; CAD: coronary artery disease; CI: confidence interval; CVA: cerebrovascular accident; CVD: cardiovascular disease; HR: hazard ratio; OR: odds ratio.

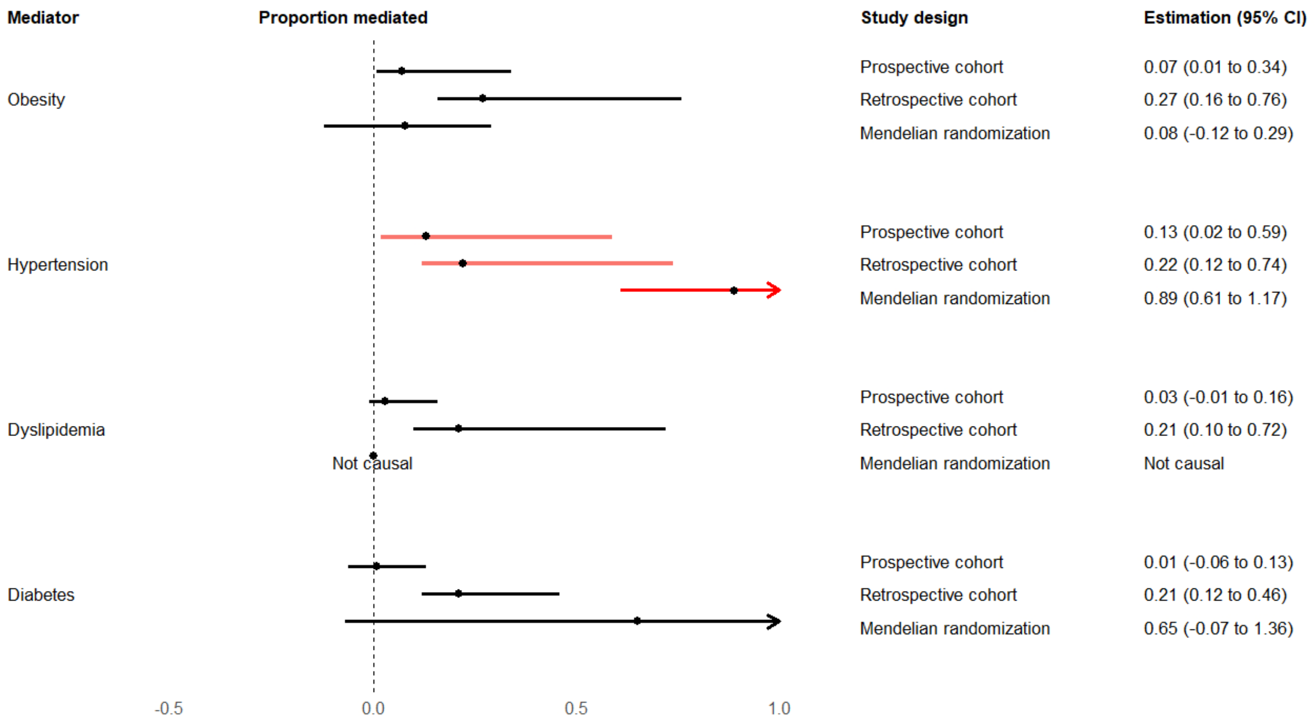

Figure S4. Proportion of the mediated effects of early menarche on the risk of coronary artery disease

Figure legend. CI: confidence interval; The proportion mediated is defined as "not causal" if the direct and indirect effects have opposite directions.

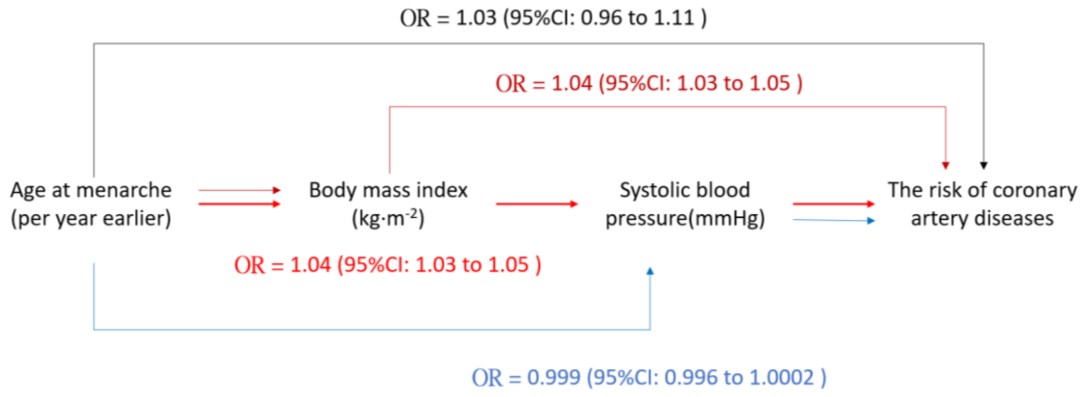

Figure S5. The pathway from age at menarche to the risk of coronary artery diseases (CAD) through body mass index (BMI) and systolic blood pressure (SBP)

Supplemental references

1. Chien KL. Mini-Review of the Chin-Shan Community Cardiovascular Cohort Study in Population Health Research in Taiwan. *Acta Cardiol Sin*. 2017;33:226-232. doi: 10.6515/acs20161021a
2. Remsberg KE, Demerath EW, Schubert CM, Chumlea WC, Sun SS, Siervogel RM. Early Menarche and the Development of Cardiovascular Disease Risk Factors in Adolescent Girls: The Fels Longitudinal Study. *The Journal of Clinical Endocrinology & Metabolism*. 2005;90:2718-2724. doi: 10.1210/jc.2004-1991 %J The Journal of Clinical Endocrinology & Metabolism
3. Lee Y, Lin RS, Sung FC, Yang C, Chien K, Chen W, Su T, Hsu H, Huang Y. Chin-Shan Community Cardiovascular Cohort in Taiwan-baseline data and five-year follow-up morbidity and mortality. *J Clin Epidemiol*. 2000;53:838-846. doi: 10.1016/s0895-4356(00)00198-0
4. VanderWeele TJ, Tchetgen Tchetgen EJ, Cornelis M, Kraft P. Methodological challenges in mendelian randomization. *Epidemiology*. 2014;25:427-435. doi: 10.1097/ede.0000000000000081
5. Burgess S, Daniel RM, Butterworth AS, Thompson SG, Consortium EP-I. Network Mendelian randomization: using genetic variants as instrumental variables to investigate mediation in causal pathways. *International journal of epidemiology*. 2015;44:484-495. doi: 10.1093/ije/dyu176
6. Carter AR, Gill D, Davies NM, Taylor AE, Tillmann T, Vaucher J, Wootton RE, Munafò MR, Hemani G, Malik R, et al. Understanding the consequences of education inequality on cardiovascular disease: mendelian randomisation study. *Bmj*. 2019;365:l1855. doi: 10.1136/bmj.l1855
7. Varbo A, Benn M, Davey Smith G, Timpson NJ, Tybjaerg-Hansen A, Nordestgaard BG. Remnant cholesterol, low-density lipoprotein cholesterol, and blood pressure as mediators from obesity to ischemic heart disease. *Circ Res*. 2015;116:665-673. doi: 10.1161/circresaha.116.304846
8. Fan HY, Huang YT, Hsieh RH, Chao JC, Tung YC, Lee YL, Chen YC. Birthweight, time-varying adiposity growth and early menarche in girls: A Mendelian randomisation and mediation analysis. *Obes Res Clin Pract*. 2018;12:445-451. doi: 10.1016/j.orcp.2018.07.008
